# *Chlamydia psittaci*: A zoonotic pathogen causing avian chlamydiosis and psittacosis

**DOI:** 10.1080/21505594.2024.2428411

**Published:** 2024-11-14

**Authors:** Jiewen Wang, Buwei Wang, Jian Xiao, Yuqing Chen, Chuan Wang

**Affiliations:** a Institute of Pathogenic Biology, School of Basic Medicine, Hengyang Medical College, University of South China; Hunan Provincial Key Laboratory for Special Pathogens Prevention and Control, Hengyang, Hunan, China; b Institute of Cell and Genetics, School of Basic Medicine, Hengyang Medical College, University of South China, Hengyang, Hunan, China; c The Affiliated Nanhua Hospital, Department of laboratory medicine, Hengyang Medical School, University of South China, Hengyang, Hunan, China; d Clinical Microbiology Laboratory, Xiangtan Central Hospital, Xiangtan, Hunan, China

**Keywords:** *Chlamydia psittaci*, host–pathogen interaction, transmission, zoonosis, epidemiology, detection

## Abstract

*Chlamydia psittaci* is an obligate intracellular gram-negative bacterium with a unique biphasic developmental cycle. It is a zoonotic pathogen with a wide range of hosts and can cause avian chlamydiosis in birds and psittacosis in humans. The pathogen is transmitted mainly through horizontal transmission between birds. Cross-species transmission sometimes occurs and human-to-human transmission has recently been confirmed. This review provides an updated overview of *C. psittaci* from the perspective of both avian chlamydiosis and psittacosis. We include the aspects of genotype, host–pathogen interaction, transmission, epidemiology, detection and diagnosis, clinical manifestation, management, and prevention, aiming to provide a basic understanding of *C. psittaci* and offer fresh insights focused on zoonosis and cross-species transmission.

## Introduction

*Chlamydia psittaci* (*C. psittaci*) is an obligate intracellular bacterium that replicates within a membrane-bound vacuole. Within the inclusion, *C. psittaci* undergoes a biphasic developmental cycle, alternating between the elementary body (EB), which ensures extracellular survival and infection, and the reticulated body (RB), which is involved in intracellular replication and growth [1,2].

Zoonotic infections due to *C. psittaci* can cause respiratory infections in both birds and humans. *C. psittaci* has a wide range of hosts and infects birds, avian species, and mammals including humans. Avian chlamydiosis (AC) is a bacterial disease of birds caused by members of the genus *Chlamydia*. Up to now, AC caused by *C. psittaci* has been documented in 467 different species of birds [3,4]. There are 17 genotypes of *C. psittaci* [5–9], all with different host preferences and virulence. The severity of infection mainly depends on the genotype of the strains and hosts involved. Treatment mainly involves antibiotics, although sometimes failing, and tetracyclines are the drugs of choice [10]. Recent studies on *C. psittaci* vaccines have made some achievements; however, commercial AC vaccines are limited. As the recorded incidence of *C. psittaci* in birds worldwide is getting higher [11,12], and numerous laboratory-confirmed cases of psittacosis in humans are being reported in a growing number of countries [13–16], it is crucial to raise public concern about this zoonotic pathogen and the potential public health risks it brings. In this review, we attempt to provide an updated overview of *C. psittaci* from the perspective of both AC and psittacosis, aiming to offer a basic understanding of *C. psittaci* and offer fresh insights focused on zoonosis and cross-species transmission.

## Molecular epidemiology

Previously, all known avian *Chlamydia* strains were assigned to the species *C. psittaci*. However, the recent discovery of atypical *Chlamydia* and the description of new Chlamydial species (*Chlamydia gallinacea*, *Chlamydia avium*, *Chlamydia ibidis*, and *Chlamydia buteonis*) in infected birds revealed that *C. psittaci* is not the only causative agent of AC [17,18]. As *C. psittaci* has been the primary organism identified in clinical cases, here we mainly discuss the cases caused by *C. psittaci* strains.

There are about 71 genome assemblies of *C. psittaci* strains uploaded to the NCBI and ENA databases [19], which include the common strains such as 6BC, WC, M56, and Mat116. Recently, several research suggested that *C. psittaci* 84/2334 belonged to *Chlamydia abortus* (*C. abortus*) [20], and moved R54 from *C. abortus* to *C. psittaci* [8]. Besides, AMK-16 strain, a newly found *C. psittaci* strain, currently cause *C. psittaci* infection in small ruminants [21].

**Figure 1.**
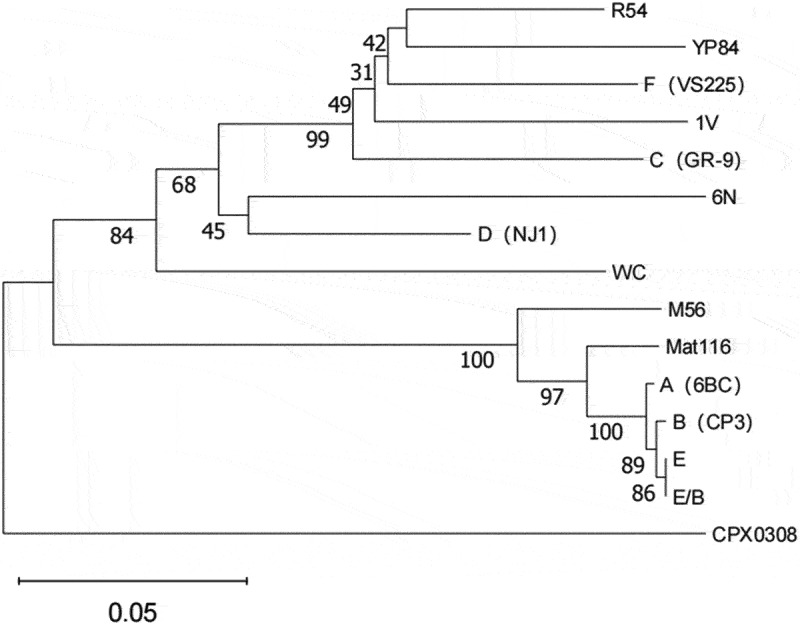
The phylogenetic tree of 15 genotypes of *C.* *psittaci*.

**Figure 2.**
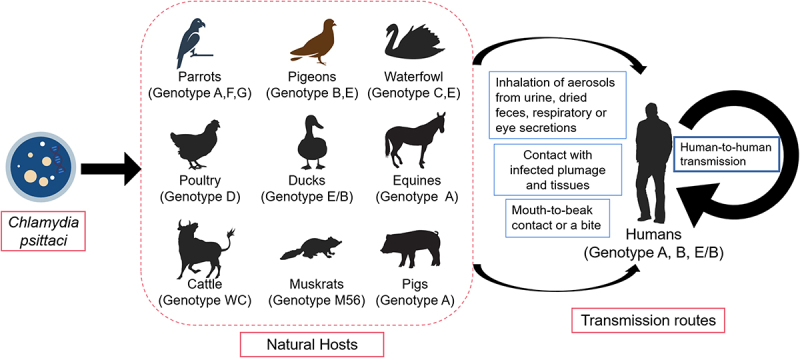
The natural hosts and transmission routes of *C.* *psittaci*.

At present, analysis of the MOMP encoding the outer membrane protein A (*ompA*) gene is generally accepted and extensively used to characterize *C. psittaci* strains into different genotypes, designated as A to G, E/B, WC, M56, 1 V, 6N, Mat116, R54, YP84, and CPX0308 (Figure 1) [22]. Some genotypes have specific preferences (Figure 2). For example, genotypes A and B occur in psittacine birds and pigeons, respectively. Genotype C is found in waterfowl, whereas genotype D is endemic in poultry (chickens and turkeys). Genotype E is primarily associated with pigeons, waterfowls, and turkeys, and genotype F tends to be associated with parakeets. Genotype E/B is found in ducks, geese, and pigeons. Genotype G was detected in red-tailed hawks, genotype WC was detected in cattle, and genotype M56 was detected in rodents. Among these, genotype A is the most common and is considered highly virulent to birds [23]. Comparative genome analysis has revealed a distinctive avian host preference of *C. psittaci* [24].

The phylogeny based on ompA gene of 15 genotypes of *C.* *psittaci*. The strains are indicated in parentheses.

The most common hosts of *C.* *psittaci* are birds, such as parrots, pigeons, turkeys, ducks, and waterfowls. *C.* *psittaci* can also infect non-avian hosts including horses, cattle, muskrats, pigs, and humans. As a zoonotic agent, the transmission of *C.* *psittaci* primarily occurs through the inhalation of contaminated aerosols from urine, dried faeces, or respiratory and eye secretions from infected animals. Meanwhile, contact with plumage and tissues of infected birds, or mouth-to-beak contact and bite from an infected bird, also contribute to chlamydial infection. Moreover, human-to-human transmission presents a new and notable zoonotic risk.

Animal infections with *C. psittaci* are distributed worldwide and have been reported in several Asian countries, South and North America, and some European and Oceania countries, while the number of epidemiological studies in Africa is limited. In China, a case characterized by a drop in egg production occurred in laying duck farms was attributed to *C. psittaci* [25]. The most common hosts of *C. psittaci* are birds, especially psittacine birds [26–30]. In European pigeons and garden birds, *C. psittaci* is abundant, as demonstrated by reports from Switzerland [31], Sweden [32], and the Netherlands [33]. In addition, this agent can infect mammals (Figure 2), such as cattle [34], equine [31,35], cats [36], and pigs [37,38]. In Australia, *C. psittaci* has been well-reported as a cause of reproductive loss in equine [35,39]. To date, the role of *C. psittaci* has been implicated as abortigenic agents in ruminants [40,41]. Interestingly, *C. psittaci* was found in a novel host, the western brush wallaby, in Australia, which was the first detection in a marsupial [42]. We speculate that such cross-species transmission may be due to co-location with other infected animals.

Although *C. psittaci* infection represents a significant economic loss to the poultry industry, it appears to be underestimated. Several studies carried out on hatcheries [43,44] have indicated that employees face a zoonotic risk and are susceptible to *C. psittaci*. Therefore, it has been proposed that *C. psittaci* should be a notifiable infectious disease and should be included in veterinary legal quarantine [12,14]. Although rare, there is a report indicating human-to-human transmission of *C. psittaci* [16]. The outbreak began with avian-to-human transmission, followed by secondary and tertiary human-to-human transmission, including multiple asymptomatic carriers and health care workers. However, numbers of infected people were limited though the bacteria spread for three generations. It seemed like the transmission ability of *C. psittaci* by human-to-human was more limited than expected.

Interestingly, there are differences in genovars in different regions (Table A1). For instance, genotype A and B seem to occur worldwide, while genotype E/B mainly appear in China in recent years [9,45]. We infer that such differences may due to geographical isolation, host populations, and migration caulting bacterial transmission (discussed in section 4 Transmission).

## Host–pathogen interaction

### Normal life cycle

Like other chlamydial species, *C. psittaci* is characterized by a biphasic developmental cycle, usually 36–72 h long. *C. psittaci* attaches to host cells in the form of EBs, the extracellular infectious form (size of 0.2 μm). After entry, endosomes containing EBs fuse to form a membrane-bound, protective intracellular replicative niche to avoid phagosome-lysosome fusion, which is termed inclusion [46]. The inclusion develops near the host nucleus, endoplasmic reticulum (ER), and Golgi apparatus to obtain raw materials such as sphingomyelin for expansion [47]. Within the inclusion, EBs differentiate into RBs, the intracellular non-infectious, but metabolically active form (size of 0.8 μm). Then, RBs replicate by binary fission in membrane-bound vacuoles using ATP and host cell metabolites. After 8–12 rounds of cell division, RBs redifferentiate into offspring EBs, exiting host cells via host lysis or extrusion. After release, mature EBs complete the developmental cycle and infect neighbouring cells.

In the early stages of the chlamydial infection cycle, adhesion to and subsequent internalization by host cells are two vital steps [46,48]. This process involves EBs. Irreversible high-affinity binding through different host receptors and bacterial ligands reversibly binds to the host cell. Chlamydial infections are initially caused by the binding of outer membrane protein OmcB as an adhesin to host cell glycosaminoglycans (GAGs) [49]. However, GAG-dependent adhesion is not the only mechanism. Recent studies have demonstrated that among the 21 Polymorphic Membrane Proteins (Pmps) of *C. psittaci*, Pmp22D, Pmp8G, and Pmp17G possess adhesive properties and activate intracellular internalization by recognizing epidermal growth factor receptor (EGFR) during infection [48,50]. In some cases, EGFR activation is required for the attachment and growth of *Chlamydia*. As an adhesin of *C. psittaci*, Pmp17G binds to multiple host cells and promotes chlamydial adhesion in an EGFR-dependent manner during early infection. More importantly, such adhesion is typically time dependent, with identifiable adhesion at 120 min after treatment with Pmp17G [48]. Moreover, it is already recognized that protein disulphide isomerase (PDI) participates in chlamydial infectivity and is necessary for entry [51].

As an intracellular pathogen, *C. psittaci* undergoes a developmental cycle in which it is confined to and parasitizes the infected host cells in membrane-bound vacuoles. At this stage, inclusion establishment is essential for intracellular survival of the pathogen. One strategy is the development of a type III secretion system (T3SS), a virulence factor. Chlamydial T3SS crosses over the inner and outer membranes as well as the plasma membrane (during host cell attachment) or inclusion membrane (during intracellular growth) [52]. T3SS consists of more than 20 proteins and is a unique mechanism that includes the translocator apparatus, effectors, and chaperones. Both T3SS-mediated activities in the early and middle cycles and late T3SS inactivation after detachment of *Chlamydia* from the inclusion membrane are central to chlamydial intracellular survival [53]. During the developmental cycle, chlamydial effector proteins are translocated into the host cell using T3SS, resulting in the disturbance of cellular proteins and the functional modulation of different host cells. The delivered proteins, termed inclusion membrane proteins (Incs), bind to the inclusion membrane and mediate crucial host–pathogen interactions [54]. Some domains of these INC proteins may be exposed into the cytoplasm and combine with molecular chaperone proteins to promote folding or delivery, ultimately manipulating host cells [55]. Research on T3SS in *C. psittaci* showed that the secretion and cellular translocation protein W (sctW) and inclusion membrane protein A (IncA) are associated with the inclusion membrane, emphasizing the influence of IncA on intracellular vesicle fusion, endocytosis, and exocytosis [52]. These proteins control the response to infection to ensure survival and development of intracellular pathogens.

Another intracellular survival strategy for *Chlamydia* is to regulate apoptosis by exploiting host cell mechanisms. Apoptosis is an essential defence mechanism against pathogens in host cells. Interestingly, *C. psittaci* can affect apoptotic pathways in a pro- or anti-apoptotic manner, depending on the cells [56]. In the early stages, *Chlamydia* inhibits apoptosis by inhibiting pro-apoptotic pathways and activating pro-survival pathways. The mechanisms of blocking apoptosis are various [54], among which some INC proteins are also essential [56]. For instance, CPSIT_0556, an INC protein of *C. psittaci*, can inhibit human polymorphonuclear neutrophil (hPMN) apoptosis through the PI3K/Akt and NF-κB pathways [57]. Another Inc protein, CPSIT_0846, has been shown to inhibit HeLa cell apoptosis [55]. However, in the middle and late stages of replication, *Chlamydia* induce apoptosis during growth and propagation [54]. It has been demonstrated that CPSIT_0842, an Inc protein of *C. psittaci*, induces macrophage apoptosis by initiating incomplete autophagy through the MAPK/ERK/mTOR signalling pathway [58].

Autophagy is an effective cellular, self-protective mechanism. Autophagy plays a dual role in host cells infected with *Chlamydia* [59]. *Chlamydia* with host cells can trigger several intracellular mechanisms that induce autophagy to promote pathogen clearance. However, as an intracellular pathogen, *Chlamydia* rely on metabolites in host cells for nutrition. Therefore, autophagy promotes the intracellular growth of *Chlamydia*. In a recent study, *C. psittaci* induced the unfolded protein response (UPR) and autophagy in human bronchial epithelial cells (HBEs) through the PERK and IRE1a signalling pathways, regulating its replication in host cells [59]. In addition, *C. psittaci* CPSIT_p7 protein was shown to induce autophagy in RAW264.7, mediated by TLR2 through the ERK signalling pathway [60].

Taken together, *C. psittaci* utilizes different strategies to ensure its intracellular survival and evade the host innate immune response, the mechanisms of which remain unknown. Therefore, further studies are needed to elucidate the mechanisms underlying chlamydial interactions with host cells.

### Persistence

Under various adverse growth environments, such as cytokine stimulation (e.g. interferon-gamma (IFN-γ)), antibiotic use (e.g. penicillin), nutritional deficiency (e.g. amino, acid, glucose, and iron deprivation), heat shock, phage infection, and viral co‑infection [47,61], developing *Chlamydia* may enter a viable but non-cultivable persistence state in stress response (variously termed aberrant bodies(ABs), persistent bodies, or chlamydial stress response), which is conducive to the immune escape of the pathogen. It is widely recognized that reduced or absent production of infectious progeny EB and the continued presence of viable organisms are two necessary conditions for chlamydial persistence [47]. During a persistent state, *Chlamydia* are unable to enter the typical development cycle, and the large, abnormal morphology and low electron density ABs within the inclusion are visible under TEM. When the stress conditions are removed, *Chlamydia* resumes the replication cycle and regenerates infectious particles.

This persistent state is often characterized by chronic, asymptomatic, or mild latent infections associated with immune escape. Unlike other bacterial pathogens, antimicrobial resistance is not a central problem in the clinical treatment of *Chlamydia* infections [62]. Conversely, *Chlamydia* persistence may lead to adverse pathological outcomes. If not eliminated, organisms may persist within the host. As the immune response weakens, persistent *Chlamydia* may reactivate, restimulate inflammation, and recruit immune effectors to the site of infection [63].

Recently, several studies have focused on the persistence of *Chlamydia* with the aim of elucidating this mechanism. Interferon (IFN)-γ is an important immunoregulatory cytokine secreted by T lymphocytes and natural killer (NK) cells and can induce *C. psittaci* persistence *in vitro*, acting as a common inducing factor for establishing *in vitro* models of the chlamydial persistence state. IFN-γ increases the activity of indoleamine-2,3-dioxygenase (IDO), resulting in the depletion of tryptophan, an essential amino acid, the lack of which probably impairs chlamydial growth. On one hand, the IDO-mediated depletion of tryptophan inhibits the replication of *Chlamydia*. However, the lack of tryptophan prevents the pathogen from differentiating into infectious EBs [54], possibly resulting in a persistent state.

Iron is already known to be a key factor for the growth and survival of *Chlamydia* and is an essential nutrient acquired from the host [64,65]. Intracellular iron stores can be decreased by IFN-γ-mediated downregulation of transferrin receptor [63]. Recently, iron was shown to reverse the growth inhibition of *C. psittaci in vitro* [66].

It has been reported that IFN-γ-induced *C. psittaci* persistence in HeLa cells results in the upregulation of 68 genes and downregulation of 109 genes [67]. The upregulated genes mostly participated in protein translation, metabolism of carbohydrates, nucleotides, and lipids, as well as general stress, whereas expression regulation and transcription, cell division and late expression, protein secretion, proteolysis and transport, membrane proteins, the tricarboxylic acid cycle, and virulence factor genes were downregulated. These results are consistent with those of another transcriptome analysis of *C. trachomatis* during persistence [68].

## Transmission

### Chlamydial shedding and environmental contamination

Some birds and mammals that cluster in large groups or live in highly dense groups provide a highly permissive environment for bacterial transmission. The migration of birds allows bacteria from different geographic regions to be introduced into new regions and populations of hosts [69]. In wild animals, faeces plays different roles, including marking territory, attracting mates, hunting prey, and avoiding predators [70]. These interactions facilitate intraspecies transmission and, more importantly, open the door to cross-species exposure.

*C. psittaci* can be excreted in faeces and nasal discharge, is resistant to drying, and remains infectious for months. Shedding may be activated by nutritional deficiencies, egg laying, breeding, crowding, chilling, and shipping [71], often intermittently, and without clinical signs [10,72]. The excretion period of *C. psittaci* during natural infection can vary depending on the virulence of the strain, the infective dose, and the host immune status. Moreover, there are significant differences in the shedding levels of organisms in indoor, paddock, and outdoor extension areas [73]. Interestingly, coinfection may exacerbate chlamydial shedding. A study in Belgian turkeys demonstrated that superinfection by *Escherichia coli* during the acute phase of *C. psittaci* infection increased *C. psittaci* excretion and stimulated chlamydial replication, indicating that the pathogenic interplay between the two could result in more severe respiratory disease [74].

Environmental pollution caused by *Chlamydia* shedding can also lead to interspecies transmission and potential zoonotic risks. A field study [75] found that *C. psittaci* infection in duck farms included horizontal and possible vertical transmission; however, environmental aspects also played an important role. The authors emphasized that contaminated soil could be an essential but underestimated transmission source, and assumed that *C. psittaci* could survive long enough in the farm environment that uninfected flocks reaching the same place (indoor, especially outdoor) would also be infected. Additionally, contaminated feed, equipment, and nesting sites are important because *C. psittaci* can survive in faeces and bedding for up to 30 days, thus generating a potential risk of transmission.

*C. psittaci* is transmitted mainly through inhalation and/or ingestion. In wild aquatic birds, contaminated water may become an infective source [69]. *C. psittaci* can be introduced into poultry when domestic poultry share aquatic or wet soil habitats with infected wild waterbirds [76]. In addition, grain-eating birds such as parrots, pheasants, pigeons, and house sparrows may be infected by inhaling contaminated grains or dust from faecal-contaminated feed-storage barns [71].

### Transmission between birds

The transmission of *C. psittaci* among birds occurs mainly through close contact between an infected bird and a susceptible bird. *C. psittaci* is found in large quantities in the respiratory secretions and faeces of infected birds [73,75]. *C. psittaci* is known to occur in 467 different species from 30 different orders of birds, including domestic, companion, and wild birds [3,4], of which pigeons and psittacine birds are the most susceptible hosts [4,10].

For birds that do not breed in dense colonies, transmission could be more easily initiated when birds congregate in large numbers during moulting, migration, or wintering [69]. *C. psittaci* can be introduced into susceptible pet birds and poultry from wild bird populations through shared ecology [71,77].

Migratory birds can carry pathogens, especially those that do not significantly affect their health status or migration [69]. As *C. psittaci* infection can be persistent, *C. psittaci*-infected birds may transmit the pathogen to other populations, which may subsequently bring *C. psittaci* to new areas.

### Vertical transmission

In addition to horizontal transmission, vertical transmission of *Chlamydia* occasionally occurs, although infrequently. Previous studies on turkeys, chickens, ducks, and sheep have shown that *C. psittaci* can be transmitted vertically [43,75,78]. During the formation of eggs in the ovaries or fallopian tubes, vertical or transovarial transmission of *C.* *psittaci* may cause infection in 1-day-old birds. Furthermore, vertical transmission has been demonstrated in parakeets, seagulls and snow geese [71]. In Australia, there were clinical cases of neonatal pneumonia and late term abortion in mares which may support in utero transmission occurrence of *C. psittaci* [79].

### Cross-species Transmission

Cross-species transmission is a significant cause of infectious diseases and poses the risk of zoonosis. With the rise of pet economy [76] and the invasion of natural habitats of wild birds by humans, the host barriers of *C. psittaci* are looser than ever before. As birds are natural hosts of *C. psittaci*, they participate effectively in the transmission and spread of the pathogen. Birds can also act as amplifying or liaison hosts for zoonotic agents with the ability to fly long distances [76]. The periodic movements of migratory birds make them potential zoonotic spreaders. The rapid spread of many migratory birds and free-living raptors makes it possible for *C. psittaci* to be transferred by the translocation of raptors or by long-distance migratory flight of birds [76,80]. Stopover sites along major flyways connect many species and populations in time and space [77]. For instance, a survey suggested that Australian parrots may have caused horse infections in Australia and potentially introduced *C. psittaci* to New Zealand [81].

Moreover, as *C. psittaci* can survive the passage through egg albumen, infected eggs from poultry farms may represent a potential source for cross-species transmission to farm workers and consumers of table eggs [82]. Furthermore, bird nests harbour various ectoparasite species that are also potential vectors of zoonotic infections. For instance, *Dermanyssus gallinae* plays a role in the spread of *C. psittaci* [76].

Human cases of infection primarily originate in birds. *C. psittaci* can be introduced into humans through direct contact with infected birds or through inhalation of infectious aerosols from faeces, urine, respiratory, and eye secretions of these birds. In some cases, contact with the plumage and tissues of infected birds, and even mouth-to-beak contact or a bite from an infected bird, also contribute to a zoonotic risk (Figure 2). Additionally, exposure to contaminated environments can result in human infections. Infection is usually underestimated, especially occupational zoonosis in psittacine keepers, poultry workers, veterinarians, and healthcare workers, and presents with inapparent symptom [43]. However, severe cases of this zoonotic disease have been well documented [83,84].

In addition, newly identified avian viruses are sometimes associated with the cross-species transmission of pathogens. A novel adenovirus detected in Mealy Parrots during a zoonotic outbreak of *C. psittaci* promoted the amplification of *C. psittaci* and transmission to humans [85]. Chlamydial load was higher in adenovirus-infected birds with a higher viral load. These findings indicate that co-infection with a novel pathogen can lead to an outbreak of *C. psittaci* infection in birds and epidemiologically linked humans.

In general, *C. psittaci* can easily and frequently cross the host barriers. The number of affected hosts and species is far more than previously estimated; therefore, there are some public health concerns. Such zoonotic risks caused by *C. psittaci* should be paid more attention.

## Detection and diagnosis

Detection of *C. psittaci* can be achieved traditionally by culture or serological tests, but is prone to false-negative results due to its low sensitivity and complex procedure. Other diagnostic tools, such as PCR-based methods, are substantially easier, faster, and more reliable than the traditional methods. Real-time PCR assay is specific, sensitive, and only takes several hours; therefore, it is performed more routinely in most diagnostic laboratories [15].

**Figure 3.**
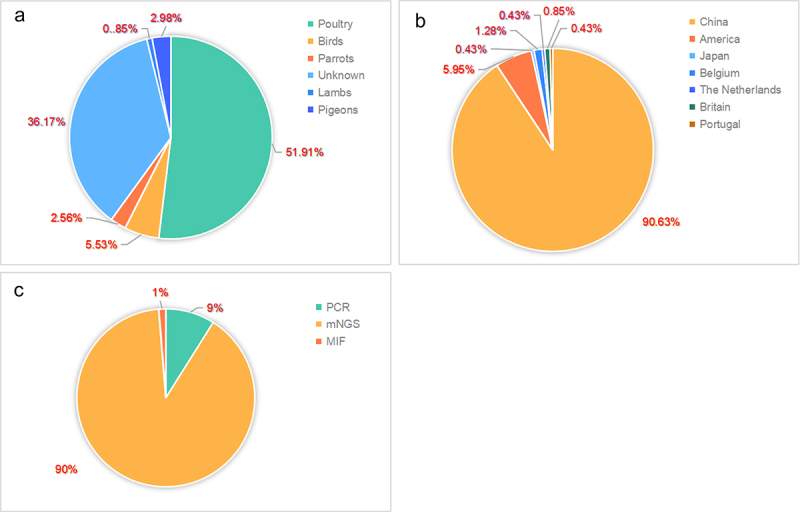
The statistic characteristics of psittacosis cases in humans in the last 5 years.

Several promising new diagnostic tools have been developed to improve the accuracy and reduce the underdiagnosis of psittacosis. For instance, metagenomic next-generation sequencing (mNGS) is available for the simultaneous detection of *C. psittaci* in the blood and bronchoalveolar lavage fluid [86–88]. According to the statistical characteristics of zoonotic cases in the past five years, the emergence of mNGS has greatly improved the diagnosis rate of *C. psittaci*, especially in China (Figure 3). Meanwhile, another study showed the advantages of mNGS in the rapid detection of *C. psittaci* [89]. Unfortunately, validation of many metagenomic-based diagnoses by confirmatory PCR testing is absent in most cases, where misdiagnosis may be triggered. Therefore, Liu et al. recommended the combination of mNGS and species-specific real-time PCR based on *ompA* for routine inclusion in the clinical diagnosis of psittacosis [14]. Interestingly, a large number of metagenome-identified cases have been found only in China. Here, we propose to promote the application of mNGS in other countries, which may improve the detection rate of the pathogen.

(a) Different sources of infection in humans. Poultry includes chicken, ducks, geese, and turkeys. Birds refer to wild birds as hosts of *C.* *psittaci* exception of parrots and pigeons. (b) The global geographical distribution of zoonotic *C.* *psittaci* cases within 5 years. (c) Diagnostic methods used for these cases.

In addition to mNGS, recombinase polymerase amplification-based assays can be used for rapid detection of *C. psittaci* in the field [90]. Furthermore, a novel, rapid, and sensitive *C. psittaci*-specific Loop-Mediated Isothermal Amplification (LAMP) was developed for detection [91].

Worryingly, there is a high rate of clinical misdiagnosis of psittacosis globally [14], due to the non-specific nature of clinical symptoms. Therefore, the differential diagnosis should be taken carefully for mycoplasma pneumonia, Legionella pneumonia, Q fever (Coxiella burnetii), influenza, brucellosis fungal pneumonia, and viral pneumonia. However, because *C. psittaci* laboratory testing is not routinely included when screening for respiratory tract infections, the pathogen is often ignored or misdiagnosed. To improve the diagnostic accuracy rate, public health workers should realize that both psittacosis and AC are not rare. Lower respiratory specimens collected shortly after symptom onset might have the highest yield for diagnosing psittacosis using real-time PCR, and stool specimens are available for the diagnosis of psittacosis [15]. Furthermore, auscultatory findings are not entirely reliable and may underestimate the extent of the pulmonary involvement [10]. Chest X-rays often show bilateral, nodular, miliary, or interstitial infiltrates or unilateral, lower-lobe dense consolidation [92].

## Clinical manifestation and treatment

The incubation period of *C. psittaci* in humans is 5–14 days^10^. *C. psittaci* mainly causes psittacosis and community-acquired pneumonia (CAP), the onset of symptoms usually appears abruptly with non-specific symptoms (flu-like symptoms) such as high fever, headache, chills, malaise, and myalgia [10]. A systematic review and meta-analysis indicated that *C. psittaci* is the causative agent of 1% of worldwide CAP [93]. However, infection with *C. psittaci* also affects other organs, including the heart, liver, spleen, joints, meninges, and central nervous system (CNS) [94]. Severe cases may develop sepsis with multiorgan failure, occasionally with fatal outcomes. Moreover, there have been several reports of pregnant women with severe *C. psittaci* infection with respiratory failure, thrombocytopenia, hepatitis, and foetal death [10,13].

Birds exposed to *C. psittaci* may exhibit acute or chronic morbid manifestations, and even death. AC usually presents with lethargy, anorexia, and ruffled feathers, similar to signs of other systemic illnesses. The severity of the disease depends on bird species, virulence of the strain, infectious dose, age, and stress factors [10].

The clinical treatment of *C. psittaci* is mainly aimed at people with psittacosis who present with pneumonia. Tetracyclines, macrolides, and quinolones can be used to treat *C. psittaci* infections (Table A1). Among these three types of antibiotics, tetracyclines are the preferred treatment for *C. psittaci* pneumonia, including tetracycline, doxycycline, and minocycline. Clinical patients with severe life-threatening conditions may require combination treatment with tetracyclines, macrolides, and quinolones [95,96]. However, a recent report revealed the use of omadacycline for the treatment of severe *C. psittaci* pneumonia in human [97]. As for gestational psittacosis, the recommended antibiotic therapy is erythromycin; other macrolides are also effective prenatally [13]. Unfortunately, no single protocol ensures safe treatment or complete elimination of infection in every bird. Therefore, treatment for AC should be supervised by a licenced veterinarian after consultation with an experienced avian veterinarian [98].

## Vaccines

Owing to latent intracellular parasitism, persistent state, and possible antibiotic resistance, the use of antibiotics cannot fundamentally control the infection. Consequently, vaccines should be emphasized as safe and effective preventive measures. To date, subunit vaccines are being studied chiefly, most of which mainly target the Major Outer Membrane Protein (MOMP), for their abundance in the outer membrane, exposed surface, and ability to elicit T-cell responses and neutralizing antibodies [63]. MOMP has emerged as the most suitable substitute for whole-cell targets, and its delivery as a combined systemic and mucosal vaccine is highly effective. However, if it is not combined with an appropriate adjuvant, MOMP may be ineffective. In a review of 220 chlamydial vaccine trials, 73 studies were adjuvant-free [99]. Interestingly, all seven successful protein-based vaccine trials used an adjuvant to stimulate immune responses during vaccination, suggesting that an adjuvant-based vaccine is essential for effective immunological response [99]. Moreover, the formulation of chlamydial inactivated antigens with adjuvants such as VCG and chitosan may also increase their ability to induce protective immune responses against challenge [100]. In a recent study, the transgenic rice seeds expressing the MOMP protein were used as an oral vaccine, and it turned out to reduce the lung lesions in mice against *C. psittaci* 6BC strain [101].

Presently, several studies have been conducted on polymorphic membrane proteins (Pmp), which are promising biomarkers. It is a cluster of surface-exposed proteins with highly conserved regions that are involved in early chlamydial infection. Polymorphic membrane protein D (PmpD) has been proven to be more valuable as it is conserved and can elicit early immune-mediated neutralization of an ongoing chlamydial infection [102–104]. A previous study has shown that a recombinant HVT vaccine expressing the N-terminal fragment of PmpD (PmpD-N) could produce a favourable protective immune response [104]. Moreover, polymorphic membrane protein G (PmpG) is also a promising vaccine candidate against Chlamydial infection. The combination of PmpG and MOMP adjuvanted with VCG and chitosan gel was proven to induce full protection both in the respiratory system and genital tract post *C. psittaci* infection [105], which might be a promising novel vaccine by blocking *C. psittaci* infection from animals to humans.

Furthermore, the chlamydial plasmid-encoded glycoprotein 3 protein (Pgp3) is also considered a promising candidate vaccine antigen [106,107], and a tandem multi-epitope vaccine based on the Pgp3 protein has been shown to possess good immunogenicity and protective efficacy against *C. psittaci* lung infection in BALB/c mice [108]. Pgp3 plays an important role in the pathogenic mechanism of *Chlamydia*. It is the main virulence factor that induce tubal effusion [107], and is able to neutralize the antichlamydial activity of the antimicrobial peptide LL 37 [106].Additionally, Pmp20G is a potential vaccine candidate against *C. psittaci* and is a highly immunogenic antigen [109–134].

Although the first recombinant MOMP vaccine for *C. psittaci* in China was registered in 2006 and commercialized for broilers [105], this commercial vaccine does not provide full protection. Therefore, further studies on more efficient and economical *C. psittaci* vaccines for avian chlamydiosis are needed and have extensive prospects for clinical applications.

## Conclusion

In summary, *C. psittaci* is a zoonotic pathogen with a wide range of hosts, and is probably underestimated by the public. The unique biphasic developmental cycle and persistent state assist in the survival and immune escape of host cells. *C. psittaci* infection resulting in AC or psittacosis is difficult to manage and is prone to misdiagnosis. Therefore, efficient detection tools such as mNGS must be developed, and effective vaccines for *C. psittaci* are urgently needed. Meanwhile, more attention should be paid to *C. psittaci* infections and the potential zoonotic risks affecting both veterinary and public health.

In the future, we propose that studies of *C. psittaci* should focus on epidemiology, to deepen our understanding of epidemiology, especially in terms of its transmission patterns and infection dynamics, as its pathogenic mechanism is not yet fully understood. Future research should explore the following directions: (i) epidemiological research: identify the epidemiological differences between *C. psittaci* and *C. trachomatis* to determine whether the detection of *C. psittaci* should be included in routine clinical practice; (ii) high sensitivity detection methods: develop and optimize the detection methods with higher sensitivity, for rapid, accurate, and convenient diagnosis of *C. psittaci* infection; (iii) cell or animal model research: evaluate the necessity of using and non-human primates as experimental models and explore more cost-effective and ethical alternative animal models to study the infection mechanism and therapeutic effects of *C. psittaci*. As birds are the natural host of *C. psittaci*, no study using bird cells to carry on research. Though the prospect, utility, and utilization value of bird cells are unclear, is there any possibility for the application of bird or avian cells in *C. psittaci* studies? (iiii) identification and functional study of virulence factors: further research should focus on the virulence factors of *C. psittaci*, including specific proteins, lipopolysaccharides, secretion systems, etc., and how they affect the bacterium’s invasive capabilities and survival within host cells. This will help reveal the specific mechanisms of how *C. psittaci* cause disease.

## Supplementary Material

Additional file 1.docx

manuscript_clean copy.docx

## Data Availability

The data used to support the findings of this study are included in the article and the supplementary information files.
