# Supplementary material for: Chlamydia psittaci: A zoonotic pathogen causing avian chlamydiosis and psittacosis
Source: Virulence. 2024 Nov 14;15(1):2428411. doi: 10.1080/21505594.2024.2428411 (PMC11622591; doi:10.1080/21505594.2024.2428411)
Supplement: Additional file 1.docx [file KVIR_A_2428411_SM2717.docx]

**Supplementary information**

**Additional File 1. The characteristics of epidemiology, diagnosis, and treatment of human *C. psittaci* infection from a clinical perspective within 5 years worldwide**

| Time | Location | Number of Confirmed Cases | Source of Infection | Genotype | Diagnostic Methods | Treatment | References |
| --- | --- | --- | --- | --- | --- | --- | --- |
| February 2018 | Fort Carson,  America | 1 | Birds | Not  determined | MIF | Levofloxacin, or doxycycline | ^110^ |
| August-October 2018 | Virginia and Georgia, America | 13 | Chicken | Not  determined | Real-time PCR | Doxycycline or macrolide antibiotic | ^15, 44^ |
| June 2018 to December 2019 | China | 15 | Unknown | Not  determined | mNGS | Not mentioned | ^89^ |
| 2019 | Belgium | 3 | Pigeons (2)  Parrots (1) | B | PCR | Doxycycline | ^111^ |
| 2019 | Flanders, Belgium | 4 | Psittacine Birds | Not  determined | PCR | Not mentioned | ^26^ |
| 2019 | Portugal | 1 | Birds | Not  determined | MIF | Doxycycline | ^112^ |
| January 2019 to November 2020 | Hunan, China | 44 | Poultry (23)  Pigeons (6)  Unknown (15) | Not  determined | mNGS | Quinolone, or  doxycycline | ^113^ |
| November 2019 to November 2020 | Hunan, China | 16 | Unknown | Not  determined | mNGS | Quinolones or minocycline | ^114^ |
| January 2019 to November 2021 | Guangdong, China | 3 | Unknown | Not  determined | mNGS | Doxycycline | ^115^ |
| December 2019 to January 2020 | Zhejiang,  China | 2 | Chicken | A | mNGS | Moxifloxacin | ^116^ |
| January 2020 | Guangdong,  China | 3 | Unknown | Not  determined | mNGS | Doxycycline | ^117^ |
| October 2020 | Hefei,  China | 1 | Poultry | Not  determined | mNGS | Doxycycline | ^118^ |
| October 2020 | Hunan,  China | 1 | Poultry | Not  determined | mNGS | Doxycycline, meropenem | ^119^ |
| October 2020 | Beijing,  China | 1 | Chicken | Not  determined | mNGS | Moxifloxacin | ^120^ |
| November 2020 | Liaoning,  China | 1 | Unknown | Not  determined | mNGS | Tigecycline | ^121^ |
| December 2020 | Beijing,  China | 1 | Chicken | Not  determined | mNGS | Minocycline, azithromycin | ^120^ |
| December 2020 | Shandong, China | 17 | Ducks | Not  determined | mNGS, qPCR | Doxycycline | ^16^ |
| December 2020 | Chongqing, China | 3 | Parrots | Not  determined | mNGS | Minocycline | ^88^ |
| January 2020 to December 2021 | Guangdong,  China | 30 | Chicken | Not  determined | mNGS | Doxycycline | ^122^ |
| April 2020 to June 2021 | Zhejiang, China | 32 | Poultry (7)  Unknown (25) | Not  determined | mNGS | Doxycycline | ^87^ |
| March 2021 | Beijing,  China | 1 | Birds | Not  determined | mNGS | Minocycline, azithromycin | ^123^ |
| June 2021 | Zhejiang, China | 1 | Chicken | Not  determined | mNGS | Doxycycline, moxifloxacin | ^124^ |
| August 2021 | Zhejiang, China | 1 | Unknown | Not  determined | mNGS | Moxifloxacin | ^125^ |
| 2021 | Guangdong,  China | 1 | Unknown | Not  determined | mNGS | Doxycycline, moxifloxacin | ^96^ |
| 2021 | Rotterdam, The Netherlands | 1 | Birds | Not  determined | PCR | Doxycycline, ciprofloxacin | ^83^ |
| 2021 | Guangdong,  China | 4 | Birds | Not  determined | mNGS | Doxycycline, or quinolones | ^84^ |
| 2021 | Hunan,  China | 5 | Poultry (3)  Unknown (2) | Not  determined | mNGS | Doxycycline | ^95^ |
| 2021 | Tokyo,  Japan | 1 | Pigeons | Not  determined | MIF | Minocycline | ^126^ |
| 2021 | Zhejiang, China | 4 | Poultry | Not  determined | mNGS | Unknown | ^127^ |
| November 2021 to January 2022 | Zhejiang, China | 4 | Geese | E/B | mNGS | Doxycycline | ^128^ |
| December 2021 to September 2022 | Zhejiang, China | 6 | Poultry (4)  Unknown (2) | Not  determined | mNGS | Doxycycline | ^129^ |
| March 2022 | Guangdong,  China | 1 | Chicken | Not  determined | mNGS | Omadacycline | ^97^ |
| April 2022 | Zhejiang,  China | 1 | Parrots | A | mNGS | Moxifloxacin, Azithromy | ^130^ |
| December 2022 | Britain | 2 | Lambs | Not  determined | mNGS | Doxycycline | ^131^ |
| 2022 | Fujian,  China | 2 | Poultry | Not  determined | mNGS | Doxycycline | ^132^ |
| 2022 | Hubei,  China | 1 | Ducks | Not  determined | mNGS | Moxifloxacin | ^133^ |
| 2022 | Hangzhou,  China | 6 | Poultry or birds (5)  Unknown (1) | B | mNGS | Doxycycline | ^134^ |
