# Supplementary material for: Chlamydia psittaci: A zoonotic pathogen causing avian chlamydiosis and psittacosis
Source: Virulence. 2024 Nov 14;15(1):2428411. doi: 10.1080/21505594.2024.2428411 (PMC11622591; doi:10.1080/21505594.2024.2428411)
Supplement: manuscript_clean copy.docx [file KVIR_A_2428411_SM2716.docx]

# *Chlamydia psittaci*: A zoonotic pathogen causing avian chlamydiosis and psittacosis

Jiewen Wang^1,2^, Buwei Wang^1^, Jian Xiao^3^, Yuqing Chen^4^, Chuan Wang^1*^

**^1^** Institute of Pathogenic Biology, School of Basic Medicine, Hengyang Medical College, University of South China; Hunan Provincial Key Laboratory for Special Pathogens Prevention and Control, University of South China, Hengyang, Hunan 421001, China

**^2^** Institute of Cell and Genetics, School of Basic Medicine, Hengyang Medical College, University of South China, Hengyang, Hunan 421001, China

**^3^** The Affiliated Nanhua Hospital, Department of laboratory medicine, Hengyang Medical School, University of South China, Hengyang, Hunan, 421001, China

**^4^** Clinical Microbiology Laboratory, Xiangtan Central Hospital, Xiangtan, Hunan, 411100, China

***Corresponding author:**

**Chuan Wang,**

Institute of Pathogenic Biology, School of Basic Medicine, Hengyang Medical College, University of South China, 28 West Changsheng Road, Hengyang, Hunan 421001, China;

Email: [wangchuan@usc.edu.cn](mailto:wangchuan@usc.edu.cn)

**Running Title:** An updated Review of *C. psittaci*

**Abstract:**

*Chlamydia psittaci* is an obligate intracellular gram-negative bacterium with a unique biphasic developmental cycle. It is a zoonotic pathogen with a wide range of hosts and can cause avian chlamydiosis in birds and psittacosis in humans. The pathogen is transmitted mainly through horizontal transmission between birds. Cross-species transmission sometimes occurs and human-to-human transmission has recently been confirmed. This review provides an updated overview of *C. psittaci* from the perspective of both avian chlamydiosis and psittacosis. We include the aspects of genotype, host-pathogen interaction, transmission, epidemiology, detection and diagnosis, clinical manifestation, management, and prevention, aiming to provide a basic understanding of *C. psittaci* and offer fresh insights focused on zoonosis and cross-species transmission.

**Keywords:** *Chlamydia psittaci*, host-pathogen interaction, transmission, zoonosis, epidemiology, detection

1. **Introduction:**

*Chlamydia psittaci* (*C. psittaci*) is an obligate intracellular bacterium that replicates within a membrane-bound vacuole. Within the inclusion, *C. psittaci* undergoes a biphasic developmental cycle, alternating between the elementary body (EB), which ensures extracellular survival and infection, and the reticulated body (RB), which is involved in intracellular replication and growth^1, 2^.

Zoonotic infections due to *C. psittaci* can cause respiratory infections in both birds and humans. *C. psittaci* has a wide range of hosts and infects birds, avian species, and mammals including humans. Avian chlamydiosis (AC) is a bacterial disease of birds caused by members of the genus *Chlamydia*. Up to now, AC caused by *C. psittaci* has been documented in 467 different species of birds^3, 4^. There are 17 genotypes of *C. psittaci*^5-9^, all with different host preferences and virulence. The severity of infection mainly depends on the genotype of the strains and hosts involved. Treatment mainly involves antibiotics, although sometimes failing, and tetracyclines are the drugs of choice^10^. Recent studies on *C. psittaci* vaccines have made some achievements; however, commercial AC vaccines are limited. As the recorded incidence of *C. psittaci* in birds worldwide is getting higher^11, 12^, and numerous laboratory-confirmed cases of psittacosis in humans are being reported in a growing number of countries^13-16^, it is crucial to raise public concern about this zoonotic pathogen and the potential public health risks it brings. In this review, we attempt to provide an updated overview of *C. psittaci* from the perspective of both AC and psittacosis, aiming to offer a basic understanding of *C. psittaci* and offer fresh insights focused on zoonosis and cross-species transmission.

1. **Molecular epidemiology ：**

Previously, all known avian *Chlamydia* strains were assigned to the species *C.* *psittaci.* However, the recent discovery of atypical *Chlamydia* and the description of new Chlamydial species (*Chlamydia gallinacea*, *Chlamydia avium*, *Chlamydia ibidis*, and *Chlamydia buteonis*) in infected birds revealed that *C. psittaci* is not the only causative agent of AC^17, 18^. As *C. psittaci* has been the primary organism identified in clinical cases, here we mainly discuss the cases caused by *C. psittaci* strains.

There are about 71 genome assemblies of *C. psittaci* strains uploaded to the NCBI and ENA databases^19^, which include the common strains such as 6BC, WC, M56, and Mat116. Recently, several research suggested that *C. psittaci* 84/2334 belonged to *Chlamydia abortus* (*C. abortus*)^20^, and moved R54 from *C. abortus* to *C. psittaci*^8^. Besides, AMK-16 strain, a newly found *C. psittaci* strain, currently cause *C.* *psittaci* infection in small ruminants^21^.

At present, analysis of the MOMP encoding the outer membrane protein A (*ompA*) gene is generally accepted and extensively used to characterize *C.* *psittaci* strains into different genotypes, designated as A to G, E/B, WC, M56, 1V, 6N, Mat116, R54, YP84, and CPX0308 (Fig. 1)^22^. Some genotypes have specific preferences (Fig. 2). For example, genotypes A and B occur in psittacine birds and pigeons, respectively. Genotype C is found in waterfowl, whereas genotype D is endemic in poultry (chickens and turkeys). Genotype E is primarily associated with pigeons, waterfowls, and turkeys, and genotype F tends to be associated with parakeets. Genotype E/B is found in ducks, geese, and pigeons. Genotype G was detected in red-tailed hawks, genotype WC was detected in cattle, and genotype M56 was detected in rodents. Among these, genotype A is the most common and is considered highly virulent to birds^23^. Comparative genome analysis has revealed a distinctive avian host preference of *C.* *psittaci*^24^.

Animal infections with *C. psittaci* are distributed worldwide and have been reported in several Asian countries, South and North America, and some European and Oceania countries, while the number of epidemiological studies in Africa is limited. In China, a case characterized by a drop in egg production occurred in laying duck farms was attributed to *C. psittaci*^25^. The most common hosts of *C.* *psittaci* are birds, especially psittacine birds^26-30^. In European pigeons and garden birds, *C. psittaci* is abundant, as demonstrated by reports from Switzerland^31^, Sweden^32^, and the Netherlands^33^. In addition, this agent can infect mammals (Fig. 2), such as cattle^34^, equine^31, 35^, cats^36^, and pigs^37, 38^. In Australia, *C. psittaci* has been well-­reported as a cause of reproductive loss in equine^35, 39^. To date, the role of *C. psittaci* has been implicated as abortigenic agents in ruminants^40, 41^. Interestingly, *C. psittaci* was found in a novel host, the western brush wallaby, in Australia, which was the first detection in a marsupial^42^. We speculate that such cross-species transmission may be due to co-location with other infected animals.

Although *C.* *psittaci* infection represents a significant economic loss to the poultry industry, it appears to be underestimated. Several studies carried out on hatcheries^43, 44^ have indicated that employees face a zoonotic risk and are susceptible to *C. psittaci*. Therefore, it has been proposed that *C. psittaci* should be a notifiable infectious disease and should be included in veterinary legal quarantine^12, 14^. Although rare, there is a report indicating human-to-human transmission of *C.* *psittaci*^16^. The outbreak began with avian-to-human transmission, followed by secondary and tertiary human-to-human transmission, including multiple asymptomatic carriers and health care workers. However, numbers of infected people were limited though the bacteria spread for three generations. It seemed like the transmission ability of *C.* *psittaci* by human-to-human was more limited than expected.

Interestingly, there are differences in genovars in different regions (Additional file 1). For instance, genotype A and B seem to occur worldwide, while genotype E/B mainly appear in China in recent years^9, 45^. We infer that such differences may due to geographical isolation, host populations, and migration caulting bacterial transmission (discussed in section 4 Transmission).

1. **Host-pathogen interaction:**

***3.1. Normal life cycle***

Like other chlamydial species, *C. psittaci* is characterized by a biphasic developmental cycle, usually 36 to 72 h long. *C. psittaci* attaches to host cells in the form of EBs, the extracellular infectious form (size of 0.2 μm). After entry, endosomes containing EBs fuse to form a membrane-bound, protective intracellular replicative niche to avoid phagosome-lysosome fusion, which is termed inclusion^46^. The inclusion develops near the host nucleus, endoplasmic reticulum (ER), and Golgi apparatus to obtain raw materials such as sphingomyelin for expansion^47^. Within the inclusion, EBs differentiate into RBs, the intracellular non-infectious, but metabolically active form (size of 0.8 μm). Then, RBs replicate by binary fission in membrane-bound vacuoles using ATP and host cell metabolites. After 8-12 rounds of cell division, RBs redifferentiate into offspring EBs, exiting host cells via host lysis or extrusion. After release, mature EBs complete the developmental cycle and infect neighboring cells.

In the early stages of the chlamydial infection cycle, adhesion to and subsequent internalization by host cells are two vital steps^46, 48^. This process involves EBs. Irreversible high-affinity binding through different host receptors and bacterial ligands reversibly binds to the host cell. Chlamydial infections are initially caused by the binding of outer membrane protein OmcB as an adhesin to host cell glycosaminoglycans (GAGs)^49^. However, GAG-dependent adhesion is not the only mechanism. Recent studies have demonstrated that among the 21 Polymorphic Membrane Proteins (Pmps) of *C. psittaci*, Pmp22D, Pmp8G, and Pmp17G possess adhesive properties and activate intracellular internalization by recognizing epidermal growth factor receptor (EGFR) during infection^48, 50^. In some cases, EGFR activation is required for the attachment and growth of *Chlamydia*. As an adhesin of *C. psittaci*, Pmp17G binds to multiple host cells and promotes chlamydial adhesion in an EGFR-dependent manner during early infection. More importantly, such adhesion is typically time dependent, with identifiable adhesion at 120 min after treatment with Pmp17G^48^. Moreover, it is already recognized that Protein Disulphide Isomerase (PDI) participates in chlamydial infectivity and is necessary for entry^51^.

As an intracellular pathogen, *C. psittaci* undergoes a developmental cycle in which it is confined to and parasitizes the infected host cells in membrane-bound vacuoles. At this stage, inclusion establishment is essential for intracellular survival of the pathogen. One strategy is the development of a type III secretion system (T3SS), a virulence factor. Chlamydial T3SS crosses over the inner and outer membranes as well as the plasma membrane (during host cell attachment) or inclusion membrane (during intracellular growth)^52^. T3SS consists of more than 20 proteins and is a unique mechanism that includes the translocator apparatus, effectors, and chaperones. Both T3SS-mediated activities in the early and middle cycles and late T3SS inactivation after detachment of *Chlamydia* from the inclusion membrane are central to chlamydial intracellular survival^53^. During the developmental cycle, chlamydial effector proteins are translocated into the host cell using T3SS, resulting in the disturbance of cellular proteins and the functional modulation of different host cells. The delivered proteins, termed inclusion membrane proteins (Incs), bind to the inclusion membrane and mediate crucial host-pathogen interactions^54^. Some domains of these INC proteins may be exposed into the cytoplasm and combine with molecular chaperone proteins to promote folding or delivery, ultimately manipulating host cells^55^. Research on T3SS in *C. psittaci* showed that the secretion and cellular translocation protein W (sctW) and inclusion membrane protein A (IncA) are associated with the inclusion membrane, emphasizing the influence of IncA on intracellular vesicle fusion, endocytosis, and exocytosis^52^. These proteins control the response to infection to ensure survival and development of intracellular pathogens.

Another intracellular survival strategy for *Chlamydia* is to regulate apoptosis by exploiting host cell mechanisms. Apoptosis is an essential defense mechanism against pathogens in host cells. Interestingly, *C. psittaci* can affect apoptotic pathways in a pro- or anti-apoptotic manner, depending on the cells^56^. In the early stages, *Chlamydia* inhibits apoptosis by inhibiting pro-apoptotic pathways and activating pro-survival pathways. The mechanisms of blocking apoptosis are various^54^, among which some INC proteins are also essential^56^. For instance, CPSIT_0556, an INC protein of *C. psittaci*, can inhibit human polymorphonuclear neutrophil (hPMN) apoptosis through the PI3K/Akt and NF-κB pathways^57^. Another Inc protein, CPSIT_0846, has been shown to inhibit HeLa cell apoptosis^55^. However, in the middle and late stages of replication, *Chlamydia* induce apoptosis during growth and propagation^54^. It has been demonstrated that CPSIT_0842, an Inc protein of *C. psittaci*, induces macrophage apoptosis by initiating incomplete autophagy through the MAPK/ERK/mTOR signaling pathway^58^.

Autophagy is an effective cellular, self-protective mechanism. Autophagy plays a dual role in host cells infected with *Chlamydia*^59^. *Chlamydia* with host cells can trigger several intracellular mechanisms that induce autophagy to promote pathogen clearance. However, as an intracellular pathogen, *Chlamydia* rely on metabolites in host cells for nutrition. Therefore, autophagy promotes the intracellular growth of *Chlamydia*. In a recent study, *C. psittaci* induced the unfolded protein response (UPR) and autophagy in human bronchial epithelial cells (HBEs) through the PERK and IRE1a signaling pathways, regulating its replication in host cells^59^. In addition, *C. psittaci* CPSIT_p7 protein was shown to induce autophagy in RAW264.7, mediated by TLR2 through the ERK signaling pathway^60^.

Taken together, *C. psittaci* utilizes different strategies to ensure its intracellular survival and evade the host innate immune response, the mechanisms of which remain unknown. Therefore, further studies are needed to elucidate the mechanisms underlying chlamydial interactions with host cells.

***3.2. Persistence***

Under various adverse growth environments, such as cytokine stimulation (e.g., interferon-gamma (IFN-γ)), antibiotic use (e.g., penicillin), nutritional deficiency (e.g., amino, acid, glucose, and iron deprivation), heat shock, phage infection, and viral co‑infection^47, 61^, developing *Chlamydia* may enter a viable but non-cultivable persistence state in stress response (variously termed aberrant bodies(ABs), persistent bodies, or chlamydial stress response), which is conducive to the immune escape of the pathogen. It is widely recognized that reduced or absent production of infectious progeny EB and the continued presence of viable organisms are two necessary conditions for chlamydial persistence^47^. During a persistent state, *Chlamydia* are unable to enter the typical development cycle, and the large, abnormal morphology and low electron density ABs within the inclusion are visible under TEM. When the stress conditions are removed, *Chlamydia* resumes the replication cycle and regenerates infectious particles.

This persistent state is often characterized by chronic, asymptomatic, or mild latent infections associated with immune escape. Unlike other bacterial pathogens, antimicrobial resistance is not a central problem in the clinical treatment of *Chlamydia* infections^62^. Conversely, *Chlamydia* persistence may lead to adverse pathological outcomes. If not eliminated, organisms may persist within the host. As the immune response weakens, persistent *Chlamydia* may reactivate, restimulate inflammation, and recruit immune effectors to the site of infection^63^.

Recently, several studies have focused on the persistence of *Chlamydia* with the aim of elucidating this mechanism. Interferon (IFN)-γ is an important immunoregulatory cytokine secreted by T lymphocytes and natural killer (NK) cells and can induce *C. psittaci* persistence *in vitro,* acting as a common inducing factor for establishing *in vitro* models of the chlamydial persistence state. IFN-γ increases the activity of indoleamine-2,3-dioxygenase (IDO), resulting in the depletion of tryptophan, an essential amino acid, the lack of which probably impairs chlamydial growth. On one hand, the IDO-mediated depletion of tryptophan inhibits the replication of *Chlamydia*. However, the lack of tryptophan prevents the pathogen from differentiating into infectious EBs^54^, possibly resulting in a persistent state.

Iron is already known to be a key factor for the growth and survival of *Chlamydia* and is an essential nutrient acquired from the host^64, 65^. Intracellular iron stores can be decreased by IFN-γ-mediated downregulation of transferrin receptor^63^. Recently, iron was shown to reverse the growth inhibition of *C. psittaci in vitro*^66^.

It has been reported that IFN-γ-induced *C. psittaci* persistence in HeLa cells results in the upregulation of 68 genes and downregulation of 109 genes^67^. The upregulated genes mostly participated in protein translation, metabolism of carbohydrates, nucleotides, and lipids, as well as general stress, whereas expression regulation and transcription, cell division and late expression, protein secretion, proteolysis and transport, membrane proteins, the tricarboxylic acid cycle, and virulence factor genes were downregulated. These results are consistent with those of another transcriptome analysis of *C. trachomatis* during persistence^68^*.*

1. **Transmission:**
   1. ***Chlamydial shedding and environmental contamination***

Some birds and mammals that cluster in large groups or live in highly dense groups provide a highly permissive environment for bacterial transmission. The migration of birds allows bacteria from different geographic regions to be introduced into new regions and populations of hosts^69^. In wild animals, feces plays different roles, including marking territory, attracting mates, hunting prey, and avoiding predators^70^. These interactions facilitate intraspecies transmission and, more importantly, open the door to cross-species exposure.

*C. psittaci* can be excreted in feces and nasal discharge, is resistant to drying, and remains infectious for months. Shedding may be activated by nutritional deficiencies, egg laying, breeding, crowding, chilling, and shipping^71^, often intermittently, and without clinical signs ^10, 72^. The excretion period of *C. psittaci* during natural infection can vary depending on the virulence of the strain, the infective dose, and the host immune status. Moreover, there are significant differences in the shedding levels of organisms in indoor, paddock, and outdoor extension areas^73^. Interestingly, coinfection may exacerbate chlamydial shedding. A study in Belgian turkeys demonstrated that superinfection by *Escherichia coli* during the acute phase of *C. psittaci* infection increased *C. psittaci* excretion and stimulated chlamydial replication, indicating that the pathogenic interplay between the two could result in more severe respiratory disease^74^.

Environmental pollution caused by *Chlamydia* shedding can also lead to interspecies transmission and potential zoonotic risks. A field study^75^ found that *C. psittaci* infection in duck farms included horizontal and possible vertical transmission; however, environmental aspects also played an important role. The authors emphasized that contaminated soil could be an essential but underestimated transmission source, and assumed that *C. psittaci* could survive long enough in the farm environment that uninfected flocks reaching the same place (indoor, especially outdoor) would also be infected. Additionally, contaminated feed, equipment, and nesting sites are important because *C. psittaci* can survive in feces and bedding for up to 30 days, thus generating a potential risk of transmission.

*C. psittaci* is transmitted mainly through inhalation and/or ingestion. In wild aquatic birds, contaminated water may become an infective source^69^. *C. psittaci* can be introduced into poultry when domestic poultry share aquatic or wet soil habitats with infected wild waterbirds^76^. In addition, grain-eating birds such as parrots, pheasants, pigeons, and house sparrows may be infected by inhaling contaminated grains or dust from fecal-contaminated feed-storage barns^71^.

***4.2. Transmission between birds***

The transmission of *C. psittaci* among birds occurs mainly through close contact between an infected bird and a susceptible bird. *C. psittaci* is found in large quantities in the respiratory secretions and feces of infected birds^73, 75^. *C. psittaci* is known to occur in 467 different species from 30 different orders of birds, including domestic, companion, and wild birds^3, 4^, of which pigeons and psittacine birds are the most susceptible hosts^4, 10^.

For birds that do not breed in dense colonies, transmission could be more easily initiated when birds congregate in large numbers during molting, migration, or wintering^69^. *C. psittaci* can be introduced into susceptible pet birds and poultry from wild bird populations through shared ecology^71, 77^.

Migratory birds can carry pathogens, especially those that do not significantly affect their health status or migration^69^. As *C. psittaci* infection can be persistent, *C. psittaci*-infected birds may transmit the pathogen to other populations, which may subsequently bring *C. psittaci* to new areas.

***4.3. Vertical transmission***

In addition to horizontal transmission, vertical transmission of Chlamydia occasionally occurs, although infrequently. Previous studies on turkeys, chickens, ducks, and sheep have shown that *C. psittaci* can be transmitted vertically^43, 75, 78^. During the formation of eggs in the ovaries or fallopian tubes, vertical or transovarial transmission of C. psittaci may cause infection in 1-day-old birds. Furthermore, vertical transmission has been demonstrated in parakeets, seagulls and snow geese^71^. In Australia, there were clinical cases of neonatal pneumonia and late term abortion in mares which may support in utero transmission occurrence of *C. psittaci*^79^.

***4.4. Cross-species Transmission***

Cross-species transmission is a significant cause of infectious diseases and poses the risk of zoonosis. With the rise of pet economy^76^ and the invasion of natural habitats of wild birds by humans, the host barriers of *C. psittaci* are looser than ever before. As birds are natural hosts of *C. psittaci*, they participate effectively in the transmission and spread of the pathogen. Birds can also act as amplifying or liaison hosts for zoonotic agents with the ability to fly long distances^76^. The periodic movements of migratory birds make them potential zoonotic spreaders. The rapid spread of many migratory birds and free-living raptors makes it possible for *C. psittaci* to be transferred by the translocation of raptors or by long-distance migratory flight of birds^76, 80^. Stopover sites along major flyways connect many species and populations in time and space^77^. For instance, a survey suggested that Australian parrots may have caused horse infections in Australia and potentially introduced *C. psittaci* to New Zealand^81^.

Moreover, as *C. psittaci* can survive the passage through egg albumen, infected eggs from poultry farms may represent a potential source for cross-species transmission to farm workers and consumers of table eggs^82^. Furthermore, bird nests harbor various ectoparasite species that are also potential vectors of zoonotic infections. For instance, *Dermanyssus gallinae* plays a role in the spread of *C. psittaci*^76^*.*

Human cases of infection primarily originate in birds. *C. psittaci* can be introduced into humans through direct contact with infected birds or through inhalation of infectious aerosols from feces, urine, respiratory, and eye secretions of these birds. In some cases, contact with the plumage and tissues of infected birds, and even mouth-to-beak contact or a bite from an infected bird, also contribute to a zoonotic risk (Fig. 2). Additionally, exposure to contaminated environments can result in human infections. Infection is usually underestimated, especially occupational zoonosis in psittacine keepers, poultry workers, veterinarians, and healthcare workers, and presents with inapparent symptom^43^. However, severe cases of this zoonotic disease have been well documented^83, 84^.

In addition, newly identified avian viruses are sometimes associated with the cross-species transmission of pathogens. A novel adenovirus detected in Mealy Parrots during a zoonotic outbreak of *C. psittaci* promoted the amplification of *C. psittaci* and transmission to humans^85^. Chlamydial load was higher in adenovirus-infected birds with a higher viral load. These findings indicate that co-infection with a novel pathogen can lead to an outbreak of *C. psittaci* infection in birds and epidemiologically linked humans.

In general, *C. psittaci* can easily and frequently cross the host barriers. The number of affected hosts and species is far more than previously estimated; therefore, there are some public health concerns. Such zoonotic risks caused by *C. psittaci* should be paid more attention.

1. **Detection and Diagnosis：**

Detection of *C. psittaci* can be achieved traditionally by culture or serological tests, but is prone to false-negative results due to its low sensitivity and complex procedure. Other diagnostic tools, such as PCR-based methods, are substantially easier, faster, and more reliable than the traditional methods. Real-time PCR assay is specific, sensitive, and only takes several hours; therefore, it is performed more routinely in most diagnostic laboratories^15^.

Several promising new diagnostic tools have been developed to improve the accuracy and reduce the underdiagnosis of psittacosis. For instance, metagenomic next-generation sequencing (mNGS) is available for the simultaneous detection of *C. psittaci* in the blood and bronchoalveolar lavage fluid^86-88^. According to the statistical characteristics of zoonotic cases in the past five years, the emergence of mNGS has greatly improved the diagnosis rate of *C. psittaci*, especially in China (Fig. 3). Meanwhile, another study showed the advantages of mNGS in the rapid detection of *C. psittaci*^89^. Unfortunately, validation of many metagenomic-based diagnoses by confirmatory PCR testing is absent in most cases, where misdiagnosis may be triggered. Therefore, Liu et al. recommended the combination of mNGS and species-specific real-time PCR based on *ompA* for routine inclusion in the clinical diagnosis of psittacosis^14^. Interestingly, a large number of metagenome-identified cases have been found only in China. Here, we propose to promote the application of mNGS in other countries, which may improve the detection rate of the pathogen.

In addition to mNGS, recombinase polymerase amplification-based assays can be used for rapid detection of *C.* *psittaci* in the field^90^. Furthermore, a novel, rapid, and sensitive *C. psittaci*-specific Loop-Mediated Isothermal Amplification (LAMP) was developed for detection^91^.

Worryingly, there is a high rate of clinical misdiagnosis of psittacosis globally^14^, due to the non-specific nature of clinical symptoms. Therefore, the differential diagnosis should be taken carefully for mycoplasma pneumonia, Legionella pneumonia, Q fever (Coxiella burnetii), influenza, brucellosis fungal pneumonia, and viral pneumonia. However, because *C. psittaci* laboratory testing is not routinely included when screening for respiratory tract infections, the pathogen is often ignored or misdiagnosed. To improve the diagnostic accuracy rate, public health workers should realize that both psittacosis and AC are not rare. Lower respiratory specimens collected shortly after symptom onset might have the highest yield for diagnosing psittacosis using real-time PCR, and stool specimens are available for the diagnosis of psittacosis^15^. Furthermore, auscultatory findings are not entirely reliable and may underestimate the extent of the pulmonary involvement^10^. Chest X-rays often show bilateral, nodular, miliary, or interstitial infiltrates or unilateral, lower-lobe dense consolidation^92^.

1. **Clinical manifestation and Treatment：**

The incubation period of *C. psittaci* in humans is 5–14 days^10^. *C. psittaci* mainly causes psittacosis and community-acquired pneumonia (CAP), the onset of symptoms usually appears abruptly with non-specific symptoms (flu-like symptoms) such as high fever, headache, chills, malaise, and myalgia^10^. A systematic review and meta-analysis indicated that *C. psittaci* is the causative agent of 1% of worldwide CAP^93^. However, infection with *C. psittaci* also affects other organs, including the heart, liver, spleen, joints, meninges, and central nervous system (CNS)^94^. Severe cases may develop sepsis with multiorgan failure, occasionally with fatal outcomes. Moreover, there have been several reports of pregnant women with severe *C. psittaci* infection with respiratory failure, thrombocytopenia, hepatitis, and fetal death^10, 13^.

Birds exposed to *C. psittaci*may exhibit acute or chronic morbid manifestations, and even death. AC usually presents with lethargy, anorexia, and ruffled feathers, similar to signs of other systemic illnesses. The severity of the disease depends on bird species, virulence of the strain, infectious dose, age, and stress factors^10^.

The clinical treatment of *C. psittaci* is mainly aimed at people with psittacosis who present with pneumonia. Tetracyclines, macrolides, and quinolones can be used to treat *C. psittaci* infections (Table 1). Among these three types of antibiotics, tetracyclines are the preferred treatment for *C. psittaci* pneumonia, including tetracycline, doxycycline, and minocycline. Clinical patients with severe life-threatening conditions may require combination treatment with tetracyclines, macrolides, and quinolones^95, 96^. However, a recent report revealed the use of omadacycline for the treatment of severe *C. psittaci* pneumonia in human^97^. As for gestational psittacosis, the recommended antibiotic therapy is erythromycin; other macrolides are also effective prenatally^13^. Unfortunately, no single protocol ensures safe treatment or complete elimination of infection in every bird. Therefore, treatment for AC should be supervised by a licensed veterinarian after consultation with an experienced avian veterinarian^98^.

1. **Vaccines：**

Owing to latent intracellular parasitism, persistent state, and possible antibiotic resistance, the use of antibiotics cannot fundamentally control the infection. Consequently, vaccines should be emphasized as safe and effective preventive measures. To date, subunit vaccines are being studied chiefly, most of which mainly target the Major Outer Membrane Protein (MOMP), for their abundance in the outer membrane, exposed surface, and ability to elicit T-cell responses and neutralizing antibodies^63^. MOMP has emerged as the most suitable substitute for whole-cell targets, and its delivery as a combined systemic and mucosal vaccine is highly effective. However, if it is not combined with an appropriate adjuvant, MOMP may be ineffective. In a review of 220 chlamydial vaccine trials, 73 studies were adjuvant-free^99^. Interestingly, all seven successful protein-based vaccine trials used an adjuvant to stimulate immune responses during vaccination, suggesting that an adjuvant-based vaccine is essential for effective immunological response^99^. Moreover, the formulation of chlamydial inactivated antigens with adjuvants such as VCG and chitosan may also increase their ability to induce protective immune responses against challenge^100^. In a recent study, the transgenic rice seeds expressing the MOMP protein were used as an oral vaccine, and it turned out to reduce the lung lesions in mice against *C. psittaci* 6BC strain^101^.

Presently, several studies have been conducted on polymorphic membrane proteins (Pmp), which are promising biomarkers. It is a cluster of surface-exposed proteins with highly conserved regions that are involved in early chlamydial infection. Polymorphic membrane protein D (PmpD) has been proven to be more valuable as it is conserved and can elicit early immune-mediated neutralization of an ongoing chlamydial infection^102-104^. A previous study has shown that a recombinant HVT vaccine expressing the N-terminal fragment of PmpD (PmpD-N) could produce a favorable protective immune response^104^. Moreover, polymorphic membrane protein G (PmpG) is also a promising vaccine candidate against Chlamydial infection. The combination of PmpG and MOMP adjuvanted with VCG and chitosan gel was proven to induce full protection both in the respiratory system and genital tract post *C. psittaci* infection^105^, which might be a promising novel vaccine by blocking *C. psittaci* infection from animals to humans.

Furthermore, the chlamydial plasmid-encoded glycoprotein 3 protein (Pgp3) is also considered a promising candidate vaccine antigen^106, 107^, and a tandem multi-epitope vaccine based on the Pgp3 protein has been shown to possess good immunogenicity and protective efficacy against *C. psittaci* lung infection in BALB/c mice^108^. Pgp3 plays an important role in the pathogenic mechanism of *Chlamydia*. It is the main virulence factor that induce tubal effusion^107^, and is able to neutralize the antichlamydial activity of the antimicrobial peptide LL 37^106^.Additionally, Pmp20G is a potential vaccine candidate against *C. psittaci* and is a highly immunogenic antigen^109^.

Although the first recombinant MOMP vaccine for *C. psittaci* in China was registered in 2006 and commercialized for broilers^105^, this commercial vaccine does not provide full protection. Therefore, further studies on more efficient and economical *C. psittaci* vaccines for avian chlamydiosis are needed and have extensive prospects for clinical applications.

1. **Conclusion:**

In summary, *C. psittaci* is a zoonotic pathogen with a wide range of hosts, and is probably underestimated by the public. The unique biphasic developmental cycle and persistent state assist in the survival and immune escape of host cells. *C. psittaci* infection resulting in AC or psittacosis is difficult to manage and is prone to misdiagnosis. Therefore, efficient detection tools such as mNGS must be developed, and effective vaccines for *C. psittaci* are urgently needed. Meanwhile, more attention should be paid to *C. psittaci* infections and the potential zoonotic risks affecting both veterinary and public health.

In the future, we propose that studies of *C. psittaci* should focus on epidemiology, to deepen our understanding of epidemiology, especially in terms of its transmission patterns and infection dynamics, as its pathogenic mechanism is not yet fully understood. Future research should explore the following directions: (i) epidemiological research: identify the epidemiological differences between *C. psittaci* and *C. trachomatis* to determine whether the detection of *C. psittaci* should be included in routine clinical practice; (ii) high sensitivity detection methods: develop and optimize the detection methods with higher sensitivity, for rapid, accurate, and convenient diagnosis of *C. psittaci* infection; (iii) cell or animal model research: Evaluate the necessity of using and non-human primates as experimental models and explore more cost-effective and ethical alternative animal models to study the infection mechanism and therapeutic effects of *C. psittaci.* As birds are the natural host of *C. psittaci*, no study using bird cells to carry on research. Though the prospect, utility, and utilization value of bird cells are unclear, is there any possibility for the application of bird or avian cells in *C. psittaci* studies? (iiii) identification and functional study of virulence factors: further research should focus on the virulence factors of *C. psittaci*, including specific proteins, lipopolysaccharides, secretion systems, etc., and how they affect the bacterium’s invasive capabilities and survival within host cells. This will help reveal the specific mechanisms of how *C. psittaci* cause disease.

**Author contribution**

Jiewen Wang and Chuan Wang were involved in the conception and design the drafting of the paper. Jiewen Wang and Buwei Wang designed figures and tables. Jian Xiao and Yuqing Chen revisid it critically for intellectual content. Chuan Wang was responsible for the final approval of the version to be published. All authors agreed to be accountable for all aspects of the work.

**Declarations**

**Ethics approval** Not applicable.

**Consent for publication** Not applicable.

**Competing interests** The authors declare no competing interests.

**Data Availability**

The data used to support the findings of this study are included in the article and the supplementary information files.

**Declaration of funding**

This work was supported by  the Hunan Provincial Key Laboratory for Special Pathogens Prevention and Control Foundation under Grant No.2014-5, the Projects of Hunan Education Authorities under Grant No.22B0417, the Hengyang Science and Technology Planning Project under Grant No.202250045337, and the Natural Science Foundation of Hunan Province under Grant Nos.2023JJ60054 and 2022JJ70126.

**References:**

1. Bastidas RJ, Elwell CA, Engel JN, Valdivia RH. Chlamydial intracellular survival strategies. Cold Spring Harb Perspect Med 2013; 3:a010256.

2. Knittler MR, Berndt A, Böcker S, Dutow P, Hänel F, Heuer D, et al. Chlamydia psittaci: new insights into genomic diversity, clinical pathology, host-pathogen interaction and anti-bacterial immunity. Int J Med Microbiol 2014; 304:877-93.

3. Borel N, Polkinghorne A, Pospischil A. A Review on Chlamydial Diseases in Animals: Still a Challenge for Pathologists? Veterinary pathology 2018; 55:374-90.

4. Kaleta EF, Taday EM. Avian host range of Chlamydophila spp. based on isolation, antigen detection and serology. Avian Pathol 2003; 32:435-61.

5. Van Lent S, Piet JR, Beeckman D, van der Ende A, Van Nieuwerburgh F, Bavoil P, et al. Full genome sequences of all nine Chlamydia psittaci genotype reference strains. J Bacteriol 2012; 194:6930-1.

6. Sachse K, Laroucau K, Hotzel H, Schubert E, Ehricht R, Slickers P. Genotyping of Chlamydophila psittaci using a new DNA microarray assay based on sequence analysis of ompA genes. BMC microbiology 2008; 8:63.

7. Szymańska-Czerwińska M, Mitura A, Niemczuk K, Zaręba K, Jodełko A, Pluta A, et al. Dissemination and genetic diversity of chlamydial agents in Polish wildfowl: Isolation and molecular characterisation of avian Chlamydia abortus strains. PloS one 2017; 12:e0174599.

8. Van Loock M, Vanrompay D, Herrmann B, Vander Stappen J, Volckaert G, Goddeeris BM, et al. Missing links in the divergence of Chlamydophila abortus from Chlamydophila psittaci. Int J Syst Evol Microbiol 2003; 53:761-70.

9. Read TD, Joseph SJ, Didelot X, Liang B, Patel L, Dean D. Comparative analysis of Chlamydia psittaci genomes reveals the recent emergence of a pathogenic lineage with a broad host range. mBio 2013; 4.

10. Compendium of measures to control Chlamydia psittaci infection among humans (psittacosis) and pet birds (avian chlamydiosis), 2000. Centers for Disease Control and Prevention. MMWR Recommendations and reports : Morbidity and mortality weekly report Recommendations and reports 2000; 49:3-17.

11. Yehia N, Salem HM, Mahmmod Y, Said D, Samir M, Mawgod SA, et al. Common viral and bacterial avian respiratory infections: an updated review. Poultry science 2023; 102:102553.

12. Zhai SL, Zhou X, Li CL, Li Y, Sun MF. Chlamydia psittaci should be included in veterinary legal quarantine everywhere. The Lancet Microbe 2023; 4:e666.

13. Tantengco OAG. Gestational psittacosis: an emerging infection. The Lancet Microbe 2022; 3:e728.

14. Liu S, Cui Z, Carr MJ, Meng L, Shi W, Zhang Z. Chlamydia psittaci should be a notifiable infectious disease everywhere. The Lancet Microbe 2023; 4:e62-e3.

15. McGovern OL, Kobayashi M, Shaw KA, Szablewski C, Gabel J, Holsinger C, et al. Use of Real-Time PCR for Chlamydia psittaci Detection in Human Specimens During an Outbreak of Psittacosis - Georgia and Virginia, 2018. MMWR Morbidity and mortality weekly report 2021; 70:505-9.

16. Zhang Z, Zhou H, Cao H, Ji J, Zhang R, Li W, et al. Human-to-human transmission of Chlamydia psittaci in China, 2020: an epidemiological and aetiological investigation. The Lancet Microbe 2022; 3:e512-e20.

17. Sachse K, Laroucau K. Two more species of Chlamydia-does it make a difference? Pathogens and disease 2015; 73:1-3.

18. Sachse K, Laroucau K, Riege K, Wehner S, Dilcher M, Creasy HH, et al. Evidence for the existence of two new members of the family Chlamydiaceae and proposal of Chlamydia avium sp. nov. and Chlamydia gallinacea sp. nov. Syst Appl Microbiol 2014; 37:79-88.

19. Sachse K, Hölzer M, Vorimore F, Barf LM, Sachse C, Laroucau K, et al. Genomic analysis of 61 Chlamydia psittaci strains reveals extensive divergence associated with host preference. BMC genomics 2023; 24:288.

20. Pannekoek Y, Dickx V, Beeckman DS, Jolley KA, Keijzers WC, Vretou E, et al. Multi locus sequence typing of Chlamydia reveals an association between Chlamydia psittaci genotypes and host species. PloS one 2010; 5:e14179.

21. Feodorova VA, Zaitsev SS, Lyapina AM, Kichemazova NV, Saltykov YV, Khizhnyakova MA, et al. Whole genome sequencing characteristics of Chlamydia psittaci caprine AMK-16 strain, a promising killed whole cell veterinary vaccine candidate against chlamydia infection. PloS one 2023; 18:e0293612.

22. Rybarczyk J, Versteele C, Lernout T, Vanrompay D. Human psittacosis: a review with emphasis on surveillance in Belgium. Acta Clin Belg 2020; 75:42-8.

23. Solorzano-Morales A, Dolz G. Molecular characterization of Chlamydia species in commercial and backyard poultry farms in Costa Rica. Epidemiology and infection 2022; 150:1-18.

24. Hölzer M, Barf LM, Lamkiewicz K, Vorimore F, Lataretu M, Favaroni A, et al. Comparative Genome Analysis of 33 Chlamydia Strains Reveals Characteristic Features of Chlamydia Psittaci and Closely Related Species. Pathogens (Basel, Switzerland) 2020; 9.

25. Lin W, Chen T, Liao L, Wang Z, Xiao J, Lu J, et al. A parrot-type Chlamydia psittaci strain is in association with egg production drop in laying ducks. Transboundary and emerging diseases 2019; 66:2002-10.

26. De Meyst A, Aaziz R, Pex J, Braeckman L, Livingstone M, Longbottom D, et al. Prevalence of New and Established Avian Chlamydial Species in Humans and Their Psittacine Pet Birds in Belgium. Microorganisms 2022; 10.

27. Tripinichgul S, Weerakhun S, Kanistanon K. Prevalence and Risk Factors of Avian Chlamydiosis Detected by Polymerase Chain Reaction in Psittacine Birds in Thailand. J Avian Med Surg 2023; 36:372-9.

28. Lee HJ, Lee OM, Kang SI, Yeo YG, Jeong JY, Kwon YK, et al. Prevalence of asymptomatic infections of Chlamydia psittaci in psittacine birds in Korea. Zoonoses Public Health 2023.

29. Muroni G, Pinna L, Serra E, Chisu V, Mandas D, Coccollone A, et al. A Chlamydia psittaci Outbreak in Psittacine Birds in Sardinia, Italy. Int J Environ Res Public Health 2022; 19.

30. Bonwitt J, Riethman M, Glashower D, Oltean HN, Wohrle R, Joseph B, et al. Application of environmental sampling to investigate a case of avian chlamydiosis in a pet store and breeding facility leading to mass bird exposures. Zoonoses Public Health 2023; 70:572-7.

31. Mattmann P, Marti H, Borel N, Jelocnik M, Albini S, Vogler BR. Chlamydiaceae in wild, feral and domestic pigeons in Switzerland and insight into population dynamics by Chlamydia psittaci multilocus sequence typing. PloS one 2019; 14:e0226088.

32. Spörndly-Nees E, Uhlhorn H, Jinnerot T, Neimanis A. Chlamydia psittaci in garden birds in Sweden. One Health 2023; 16:100542.

33. Burt SA, Röring RE, Heijne M. Chlamydia psittaci and C. avium in feral pigeon (Columba livia domestica) droppings in two cities in the Netherlands. The veterinary quarterly 2018; 38:63-6.

34. Li J, Guo W, Kaltenboeck B, Sachse K, Yang Y, Lu G, et al. Chlamydia pecorum is the endemic intestinal species in cattle while C. gallinacea, C. psittaci and C. pneumoniae associate with sporadic systemic infection. Veterinary microbiology 2016; 193:93-9.

35. Jenkins C, Jelocnik M, Micallef ML, Galea F, Taylor-Brown A, Bogema DR, et al. An epizootic of Chlamydia psittaci equine reproductive loss associated with suspected spillover from native Australian parrots. Emerging microbes & infections 2018; 7:88.

36. Sanderson H, Vasquez M, Killion H, Vance M, Sondgeroth K, Fox J. Fatal Chlamydia psittaci infection in a domestic kitten. Journal of veterinary diagnostic investigation : official publication of the American Association of Veterinary Laboratory Diagnosticians, Inc 2021; 33:101-3.

37. Sheng CY, Gong QL, Ma BY, Liu Y, Ge GY, Li DL, et al. Prevalence of Chlamydia in Pigs in China from 1985 to 2020: A Systematic Review and Meta-Analysis. Vector Borne Zoonotic Dis 2021; 21:517-33.

38. Schautteet K, Vanrompay D. Chlamydiaceae infections in pig. Veterinary research 2011; 42:29.

39. White RT, Anstey SI, Kasimov V, Jenkins C, Devlin J, El-Hage C, et al. One clone to rule them all: Culture-independent genomics of Chlamydia psittaci from equine and avian hosts in Australia. Microb Genom 2022; 8.

40. Lenzko H, Moog U, Henning K, Lederbach R, Diller R, Menge C, et al. High frequency of chlamydial co-infections in clinically healthy sheep flocks. BMC Vet Res 2011; 7:29.

41. Berri M, Rekiki A, Boumedine KS, Rodolakis A. Simultaneous differential detection of Chlamydophila abortus, Chlamydophila pecorum and Coxiella burnetii from aborted ruminant's clinical samples using multiplex PCR. BMC microbiology 2009; 9:130.

42. Anstey SI, Kasimov V, Jenkins C, Legione A, Devlin J, Amery-Gale J, et al. Chlamydia Psittaci ST24: Clonal Strains of One Health Importance Dominate in Australian Horse, Bird and Human Infections. Pathogens (Basel, Switzerland) 2021; 10.

43. Dickx V, Vanrompay D. Zoonotic transmission of Chlamydia psittaci in a chicken and turkey hatchery. Journal of medical microbiology 2011; 60:775-9.

44. Shaw KA, Szablewski CM, Kellner S, Kornegay L, Bair P, Brennan S, et al. Psittacosis Outbreak among Workers at Chicken Slaughter Plants, Virginia and Georgia, USA, 2018. Emerg Infect Dis 2019; 25:2143-5.

45. Akter R, Sansom FM, El-Hage CM, Gilkerson JR, Legione AR, Devlin JM. A 25-year retrospective study of Chlamydia psittaci in association with equine reproductive loss in Australia. Journal of medical microbiology 2021; 70.

46. Escalante-Ochoa C, Ducatelle R, Haesebrouck F. The intracellular life of Chlamydia psittaci: how do the bacteria interact with the host cell? FEMS Microbiol Rev 1998; 22:65-78.

47. Schoborg RV. Chlamydia persistence -- a tool to dissect chlamydia--host interactions. Microbes and infection 2011; 13:649-62.

48. Li X, Zuo Z, Wang Y, Hegemann JH, He C. Polymorphic Membrane Protein 17G of Chlamydia psittaci Mediated the Binding and Invasion of Bacteria to Host Cells by Interacting and Activating EGFR of the Host. Frontiers in immunology 2021; 12:818487.

49. Moelleken K, Hegemann JH. The Chlamydia outer membrane protein OmcB is required for adhesion and exhibits biovar-specific differences in glycosaminoglycan binding. Molecular microbiology 2008; 67:403-19.

50. Favaroni A, Trinks A, Weber M, Hegemann JH, Schnee C. Pmp Repertoires Influence the Different Infectious Potential of Avian and Mammalian Chlamydia psittaci Strains. Frontiers in microbiology 2021; 12:656209.

51. Abromaitis S, Stephens RS. Attachment and entry of Chlamydia have distinct requirements for host protein disulfide isomerase. PLoS pathogens 2009; 5:e1000357.

52. Beeckman DS, Geens T, Timmermans JP, Van Oostveldt P, Vanrompay DC. Identification and characterization of a type III secretion system in Chlamydophila psittaci. Veterinary research 2008; 39:27.

53. Peters J, Wilson DP, Myers G, Timms P, Bavoil PM. Type III secretion à la Chlamydia. Trends in microbiology 2007; 15:241-51.

54. Chen H, Wen Y, Li Z. Clear Victory for Chlamydia: The Subversion of Host Innate Immunity. Frontiers in microbiology 2019; 10:1412.

55. Tang T, Wu H, Chen X, Chen L, Liu L, Li Z, et al. The Hypothetical Inclusion Membrane Protein CPSIT_0846 Regulates Mitochondrial-Mediated Host Cell Apoptosis via the ERK/JNK Signaling Pathway. Frontiers in cellular and infection microbiology 2021; 11:607422.

56. Li L, Wang C, Wen Y, Hu Y, Xie Y, Xu M, et al. ERK1/2 and the Bcl-2 Family Proteins Mcl-1, tBid, and Bim Are Involved in Inhibition of Apoptosis During Persistent Chlamydia psittaci Infection. Inflammation 2018; 41:1372-83.

57. He Z, Xiao J, Wang J, Lu S, Zheng K, Yu M, et al. The Chlamydia psittaci Inclusion Membrane Protein 0556 Inhibits Human Neutrophils Apoptosis Through PI3K/AKT and NF-κB Signaling Pathways. Frontiers in immunology 2021; 12:694573.

58. Huang Y, Li S, He S, Li Y, He Q, Wu Y. Chlamydia psittaci inclusion membrane protein CPSIT_0842 induces macrophage apoptosis through MAPK/ERK-mediated autophagy. Int J Biochem Cell Biol 2023; 157:106376.

59. Chen L, Huang Q, Bai Q, Tong T, Zhou Y, Li Z, et al. Chlamydia psittaci Induces Autophagy in Human Bronchial Epithelial Cells via PERK and IRE1α, but Not ATF6 Pathway. Infect Immun 2022; 90:e0007922.

60. Luo Y, Sun Z, Chen Q, Xiao J, Yan X, Li Y, et al. TLR2 mediates autophagy through ERK signaling pathway in Chlamydia psittaci CPSIT_p7 protein-stimulated RAW264.7 cells. Microbiol Immunol 2023.

61. Chen Z, Chen L, Wang C, Yu J, Bai Q, Yu M, et al. Transcription of seven genes in a model of interferon‑γ-induced persistent Chlamydia psittaci infection. Molecular medicine reports 2017; 16:4835-42.

62. Panzetta ME, Valdivia RH, Saka HA. Chlamydia Persistence: A Survival Strategy to Evade Antimicrobial Effects in-vitro and in-vivo. Frontiers in microbiology 2018; 9:3101.

63. Roan NR, Starnbach MN. Immune-mediated control of Chlamydia infection. Cellular microbiology 2008; 10:9-19.

64. Stelzner K, Vollmuth N, Rudel T. Intracellular lifestyle of Chlamydia trachomatis and host-pathogen interactions. Nat Rev Microbiol 2023.

65. Paradkar PN, De Domenico I, Durchfort N, Zohn I, Kaplan J, Ward DM. Iron depletion limits intracellular bacterial growth in macrophages. Blood 2008; 112:866-74.

66. He QZ, Zeng HC, Huang Y, Hu YQ, Wu YM. The type III secretion system (T3SS) of Chlamydophila psittaci is involved in the host inflammatory response by activating the JNK/ERK signaling pathway. Biomed Res Int 2015; 2015:652416.

67. Chen Y, Wang C, Mi J, Zhou Z, Wang J, Tang M, et al. Characterization and comparison of differentially expressed genes involved in Chlamydia psittaci persistent infection in vitro and in vivo. Veterinary microbiology 2021; 255:108960.

68. Belland RJ, Nelson DE, Virok D, Crane DD, Hogan D, Sturdevant D, et al. Transcriptome analysis of chlamydial growth during IFN-gamma-mediated persistence and reactivation. Proceedings of the National Academy of Sciences of the United States of America 2003; 100:15971-6.

69. Olsen B, Munster VJ, Wallensten A, Waldenström J, Osterhaus AD, Fouchier RA. Global patterns of influenza a virus in wild birds. Science 2006; 312:384-8.

70. Roach SN, Langlois RA. Intra- and Cross-Species Transmission of Astroviruses. Viruses 2021; 13.

71. Harkinezhad T, Geens T, Vanrompay D. Chlamydophila psittaci infections in birds: a review with emphasis on zoonotic consequences. Veterinary microbiology 2009; 135:68-77.

72. Magnino S, Haag-Wackernagel D, Geigenfeind I, Helmecke S, Dovc A, Prukner-Radovcić E, et al. Chlamydial infections in feral pigeons in Europe: Review of data and focus on public health implications. Veterinary microbiology 2009; 135:54-67.

73. Hulin V, Bernard P, Vorimore F, Aaziz R, Cléva D, Robineau J, et al. Assessment of Chlamydia psittaci Shedding and Environmental Contamination as Potential Sources of Worker Exposure throughout the Mule Duck Breeding Process. Applied and environmental microbiology 2015; 82:1504-18.

74. Van Loock M, Loots K, Van Heerden M, Vanrompay D, Goddeeris BM. Exacerbation of Chlamydophila psittaci pathogenicity in turkeys superinfected by Escherichia coli. Veterinary research 2006; 37:745-55.

75. Vorimore F, Thébault A, Poisson S, Cléva D, Robineau J, de Barbeyrac B, et al. Chlamydia psittaci in ducks: a hidden health risk for poultry workers. Pathogens and disease 2015; 73:1-9.

76. Contreras A, Gómez-Martín A, Paterna A, Tatay-Dualde J, Prats-Van Der Ham M, Corrales JC, et al. Epidemiological role of birds in the transmission and maintenance of zoonoses. Revue scientifique et technique (International Office of Epizootics) 2016; 35:845-62.

77. Dickx V, Kalmar ID, Tavernier P, Vanrompay D. Prevalence and genotype distribution of Chlamydia psittaci in feral Canada geese (Branta canadensis) in Belgium. Vector Borne Zoonotic Dis 2013; 13:382-4.

78. Barkallah M, Jribi H, Ben Slima A, Gharbi Y, Mallek Z, Gautier M, et al. Molecular prevalence of Chlamydia and Chlamydia-like bacteria in Tunisian domestic ruminant farms and their influencing risk factors. Transboundary and emerging diseases 2018; 65:e329-e38.

79. Anstey S, Lizárraga D, Nyari S, Chalmers G, Carrick J, Chicken C, et al. Epidemiology of Chlamydia psittaci infections in pregnant Thoroughbred mares and foals. Vet J 2021; 273:105683.

80. Schettler E, Fickel J, Hotzel H, Sachse K, Streich WJ, Wittstatt U, et al. Newcastle disease virus and Chlamydia psittaci in free-living raptors from eastern Germany. Journal of wildlife diseases 2003; 39:57-63.

81. Kasimov V, White RT, Foxwell J, Jenkins C, Gedye K, Pannekoek Y, et al. Whole-genome sequencing of Chlamydia psittaci from Australasian avian hosts: A genomics approach to a pathogen that still ruffles feathers. Microb Genom 2023; 9.

82. Ahmed B, De Boeck C, Dumont A, Cox E, De Reu K, Vanrompay D. First Experimental Evidence for the Transmission of Chlamydia psittaci in Poultry through Eggshell Penetration. Transboundary and emerging diseases 2017; 64:167-70.

83. Meijer R, van Biezen P, Prins G, Boiten HJ. Multi-organ failure with necrotic skin lesions due to infection with Chlamydia psittaci. International journal of infectious diseases : IJID : official publication of the International Society for Infectious Diseases 2021; 106:262-4.

84. Zhang A, Xia X, Yuan X, Liu Y, Niu H, Zhang Y, et al. Severe Chlamydia psittaci Pneumonia Complicated by Rhabdomyolysis: A Case Series. Infection and drug resistance 2022; 15:873-81.

85. Chan JF, To KK, Chen H, Yuen KY. Cross-species transmission and emergence of novel viruses from birds. Curr Opin Virol 2015; 10:63-9.

86. Chen X, Cao K, Wei Y, Qian Y, Liang J, Dong D, et al. Metagenomic next-generation sequencing in the diagnosis of severe pneumonias caused by Chlamydia psittaci. Infection 2020; 48:535-42.

87. Yin Q, Li Y, Pan H, Hui T, Yu Z, Wu H, et al. Atypical pneumonia caused by Chlamydia psittaci during the COVID-19 pandemic. International journal of infectious diseases : IJID : official publication of the International Society for Infectious Diseases 2022; 122:622-7.

88. Li N, Li S, Tan W, Wang H, Xu H, Wang D. Metagenomic next-generation sequencing in the family outbreak of psittacosis: the first reported family outbreak of psittacosis in China under COVID-19. Emerging microbes & infections 2021; 10:1418-28.

89. Qu J, Zhang J, Chen Y, Huang Y, Xie Y, Zhou M, et al. Aetiology of severe community acquired pneumonia in adults identified by combined detection methods: a multi-centre prospective study in China. Emerging microbes & infections 2022; 11:556-66.

90. Pang Y, Cong F, Zhang X, Li H, Chang YF, Xie Q, et al. A recombinase polymerase amplification-based assay for rapid detection of Chlamydia psittaci. Poultry science 2021; 100:585-91.

91. Jelocnik M, Islam MM, Madden D, Jenkins C, Branley J, Carver S, et al. Development and evaluation of rapid novel isothermal amplification assays for important veterinary pathogens: Chlamydia psittaci and Chlamydia pecorum. PeerJ 2017; 5:e3799.

92. Beeckman DS, Vanrompay DC. Zoonotic Chlamydophila psittaci infections from a clinical perspective. Clinical microbiology and infection : the official publication of the European Society of Clinical Microbiology and Infectious Diseases 2009; 15:11-7.

93. Hogerwerf L, B DEG, Baan B, W VDH. Chlamydia psittaci (psittacosis) as a cause of community-acquired pneumonia: a systematic review and meta-analysis. Epidemiology and infection 2017; 145:3096-105.

94. Li Y, Lin F, Li W, Chen G, Li S, Liu B, et al. Comparison of clinical, laboratory and radiological characteristics between Chlamydia psittaci and adenovirus pneumonias: a multicenter retrospective study. International journal of infectious diseases : IJID : official publication of the International Society for Infectious Diseases 2023; 126:114-24.

95. Teng XQ, Gong WC, Qi TT, Li GH, Qu Q, Lu Q, et al. Clinical Analysis of Metagenomic Next-Generation Sequencing Confirmed Chlamydia psittaci Pneumonia: A Case Series and Literature Review. Infection and drug resistance 2021; 14:1481-92.

96. Shi Y, Chen J, Shi X, Hu J, Li H, Li X, et al. A case of chlamydia psittaci caused severe pneumonia and meningitis diagnosed by metagenome next-generation sequencing and clinical analysis: a case report and literature review. BMC infectious diseases 2021; 21:621.

97. Fang C, Xu L, Tan J, Tan H, Lin J, Zhao Z. Omadacycline for the Treatment of Severe Chlamydia psittaci Pneumonia Complicated with Multiple Organ Failure: A Case Report. Infection and drug resistance 2022; 15:5831-8.

98. Balsamo G, Maxted AM, Midla JW, Murphy JM, Wohrle R, Edling TM, et al. Compendium of Measures to Control Chlamydia psittaci Infection Among Humans (Psittacosis) and Pet Birds (Avian Chlamydiosis), 2017. J Avian Med Surg 2017; 31:262-82.

99. Phillips S, Quigley BL, Timms P. Seventy Years of Chlamydia Vaccine Research - Limitations of the Past and Directions for the Future. Frontiers in microbiology 2019; 10:70.

100. Zuo Z, Zou Y, Li Q, Guo Y, Zhang T, Wu J, et al. Intranasal immunization with inactivated chlamydial elementary bodies formulated in VCG-chitosan nanoparticles induces robust immunity against intranasal Chlamydia psittaci challenge. Scientific reports 2021; 11:10389.

101. Zhang XX, Yu H, Wang XH, Li XZ, Zhu YP, Li HX, et al. Protective efficacy against Chlamydophila psittaci by oral immunization based on transgenic rice expressing MOMP in mice. Vaccine 2013; 31:698-703.

102. Crane DD, Carlson JH, Fischer ER, Bavoil P, Hsia RC, Tan C, et al. Chlamydia trachomatis polymorphic membrane protein D is a species-common pan-neutralizing antigen. Proceedings of the National Academy of Sciences of the United States of America 2006; 103:1894-9.

103. Wehrl W, Brinkmann V, Jungblut PR, Meyer TF, Szczepek AJ. From the inside out--processing of the Chlamydial autotransporter PmpD and its role in bacterial adhesion and activation of human host cells. Molecular microbiology 2004; 51:319-34.

104. Liu S, Sun W, Huang X, Zhang W, Jia C, Luo J, et al. A Promising Recombinant Herpesvirus of Turkeys Vaccine Expressing PmpD-N of Chlamydia psittaci Based on Elongation Factor-1 Alpha Promoter. Frontiers in veterinary science 2017; 4:221.

105. Li Q, Chen S, Yan Z, Fang H, Wang Z, He C. A Novel Intranasal Vaccine With PmpGs + MOMP Induces Robust Protections Both in Respiratory Tract and Genital System Posting Chlamydia psittaci Infection. Frontiers in veterinary science 2022; 9:855447.

106. Hou S, Sun X, Dong X, Lin H, Tang L, Xue M, et al. Chlamydial plasmid-encoded virulence factor Pgp3 interacts with human cathelicidin peptide LL-37 to modulate immune response. Microbes and infection 2019; 21:50-5.

107. Peng B, Zhong S, Hua Y, Luo Q, Dong W, Wang C, et al. Efficacy of Pgp3 vaccination for Chlamydia urogenital tract infection depends on its native conformation. Frontiers in immunology 2022; 13:1018774.

108. Wang C, Li Y, Wang S, Yan X, Xiao J, Chen Y, et al. Evaluation of a tandem Chlamydia psittaci Pgp3 multiepitope peptide vaccine against a pulmonary chlamydial challenge in mice. Microbial pathogenesis 2020; 147:104256.

109. Cui L, Qu G, Chen Y, Wu Y, Wang C, Cheng H, et al. Polymorphic membrane protein 20G: A promising diagnostic biomarker for specific detection of Chlamydia psittaci infection. Microbial pathogenesis 2021; 155:104882.

110. Stidham RA, Richmond-Haygood M. Case report: Possible psittacosis in a military family member-clinical and public health management issues in military settings. Msmr 2019; 26:2-7.

111. Missault S, De Meyst A, Van Elslande J, Van den Abeele AM, Steen E, Van Acker J, et al. Three Cases of Atypical Pneumonia with Chlamydia psittaci: The Role of Laboratory Vigilance in the Diagnosis of Psittacosis. Pathogens (Basel, Switzerland) 2022; 12.

112. Cipriano A, Machado A, Santos FV, Abreu MA, Castro RS. [Human Psittacosis: A Case Report]. Acta Med Port 2019; 32:161-4.

113. Li H, Hao B, Wang Y, Yu D, Chen Z, Du D, et al. Metagenomic next-generation sequencing for the diagnosis of Chlamydia psittaci pneumonia. Clin Respir J 2022; 16:513-21.

114. Xiao Q, Shen W, Zou Y, Dong S, Tan Y, Zhang X, et al. Sixteen cases of severe pneumonia caused by Chlamydia psittaci in South China investigated via metagenomic next-generation sequencing. Journal of medical microbiology 2021; 70.

115. Zhang A, Xia X, Yuan X, Lv Y, Liu Y, Niu H, et al. Clinical characteristics of 14 cases of severe Chlamydia psittaci pneumonia diagnosed by metagenomic next-generation sequencing: A case series. Medicine 2022; 101:e29238.

116. Liu S, Yang Y, Pang Z, Liu Y, Li H, Cai J, et al. A cluster of two psittacosis cases among women farmers exposed to Chlamydia psittaci-infected domestic poultry in Zhejiang Province, China. Zoonoses Public Health 2023; 70:93-102.

117. Zhang H, Zhan D, Chen D, Huang W, Yu M, Li Q, et al. Next-generation sequencing diagnosis of severe pneumonia from fulminant psittacosis with multiple organ failure: a case report and literature review. Ann Transl Med 2020; 8:401.

118. Sun L, Li P, Pang B, Wu P, Wang R. Gestational Psittacosis With Secondary Hemophagocytic Syndrome: A Case Report and Literature Review. Front Med (Lausanne) 2021; 8:755669.

119. Yuan Y, Zhang X, Gui C. Detection of Chlamydia psittaci in both blood and bronchoalveolar lavage fluid using metagenomic next-generation sequencing: A case report. Medicine 2021; 100:e26514.

120. Dai N, Li Q, Geng J, Guo W, Yan W. Severe pneumonia caused by Chlamydia psittaci: report of two cases and literature review. J Infect Dev Ctries 2022; 16:1101-12.

121. Liu J, Gao Y. Tigecycline in the treatment of severe pneumonia caused by Chlamydia psittaci: A case report and literature review. Front Med (Lausanne) 2022; 9:1040441.

122. Liang Y, Dong T, Li M, Zhang P, Wei X, Chen H, et al. Clinical diagnosis and etiology of patients with Chlamydia psittaci pneumonia based on metagenomic next-generation sequencing. Frontiers in cellular and infection microbiology 2022; 12:1006117.

123. Zhou X, Bai G, Dong L, Zhuang H, Duan M. Successful Treatment of Severe Community-Acquired Pneumonia caused by Chlamydia Psittaci: a Case Report. Clin Lab 2022; 68.

124. Wang J, Zhu Y, Mo Q, Yang Y. Case Report: A Chlamydia psittaci pulmonary infection presenting with migratory infiltrates. Front Public Health 2022; 10:1028989.

125. Gao Y, Zhang X, Liu J, Gong L, Chen G, Zhou X. Chlamydia psittaci pneumonia complicated with organizing pneumonia: A case report and literature review. IDCases 2022; 30:e01584.

126. Fukui S, Kawamura W, Uehara Y, Naito T. A patient with psittacosis from a pigeon: A reminder of the importance of detailed interviews and relative bradycardia. IDCases 2021; 25:e01164.

127. Yao W, Chen X, Wu Z, Wang L, Shi G, Yang Z, et al. A cluster of Psittacosis cases in Lishui, Zhejiang Province, China, in 2021. Frontiers in cellular and infection microbiology 2022; 12:1044984.

128. Qin XC, Huang J, Yang Z, Sun X, Wang W, Gong E, et al. Severe community-acquired pneumonia caused by Chlamydia psittaci genotype E/B strain circulating among geese in Lishui city, Zhejiang province, China. Emerging microbes & infections 2022; 11:2715-23.

129. Dai J, Lian X, Mo J, Li X, Mo W, Wang H, et al. Case report: A clinical case study of six patients with Chlamydia psittaci pneumonia. Frontiers in cellular and infection microbiology 2023; 13:1084882.

130. Yao W, Yang X, Shi J, Yang Z, Yao Y, Kou J, et al. Case Report: A case of Chlamydia psittaci infection in an HIV patient. Frontiers in cellular and infection microbiology 2023; 13:1185803.

131. Guscoth LB, Taylor DM, Coad F. Persistent renal replacement requirement following fulminant psittacosis infection in pregnancy. BMJ Case Rep 2022; 15.

132. Luo C, Lin Y, Chen C, Liu Y, Sun X. Diagnosis of severe Chlamydia psittaci pneumonia by metagenomic next-generation sequencing: 2 case reports. Respir Med Case Rep 2022; 38:101709.

133. Deng F, Lin Q, Xu X, Li C, Xu J, Nie H. A case report of healthcare-associated psittacosis. J Infect Dev Ctries 2023; 17:571-7.

134. Zhu Z, Wang X, Zhao J, Xie Z, Yang C, Li L, et al. Clinical Characteristics of Six Patients with Chlamydia psittaci Infection Diagnosed by Metagenomic Next-Generation Sequencing: A Case Series. Infection and drug resistance 2023; 16:869-78.

**Figure 1. The Phylogenetic Tree of 15 genotypes of *C. psittaci.***

The phylogeny based on *ompA* gene of 15 genotypes of *C. psittaci*. The strains are indicated in parentheses.

**Figure 2. The natural hosts and transmission routes of *C. psittaci.***

The most common hosts of *C. psittaci* are birds, such as parrots, pigeons, turkeys, ducks, and waterfowls. *C. psittaci* can also infect non-avian hosts including horses, cattle, muskrats, pigs, and humans. As a zoonotic agent, the transmission of *C. psittaci* primarily occurs through the inhalation of contaminated aerosols from urine, dried feces, or respiratory and eye secretions from infected animals. Meanwhile, contact with plumage and tissues of infected birds, or mouth-to-beak contact and bite from an infected bird, also contribute to chlamydial infection. Moreover, human-to-human transmission presents a new and notable zoonotic risk.

**Figure 3. The statistic characteristics of psittacosis cases in humans in the last 5 years.**

1. Different sources of infection in humans. Poultry includes chicken, ducks, geese, and turkeys. Birds refer to wild birds as hosts of *C. psittaci* exception of parrots and pigeons. (B) The global geographical distribution of zoonotic *C. psittaci* cases within 5 years. (C) Diagnostic methods used for these cases. (Reference: Additional File 1)

**Supplementary information**

**Additional File 1. The characteristics of epidemiology, diagnosis, and treatment of human *C. psittaci* infection from a clinical perspective within 5 years worldwide**

| Time | Location | Number of Confirmed Cases | Source of Infection | Genotype | Diagnostic Methods | Treatment | References |
| --- | --- | --- | --- | --- | --- | --- | --- |
| February 2018 | Fort Carson,  America | 1 | Birds | Not  determined | MIF | Levofloxacin, or doxycycline | ^110^ |
| August-October 2018 | Virginia and Georgia, America | 13 | Chicken | Not  determined | Real-time PCR | Doxycycline or macrolide antibiotic | ^15, 44^ |
| June 2018 to December 2019 | China | 15 | Unknown | Not  determined | mNGS | Not mentioned | ^89^ |
| 2019 | Belgium | 3 | Pigeons (2)  Parrots (1) | B | PCR | Doxycycline | ^111^ |
| 2019 | Flanders, Belgium | 4 | Psittacine Birds | Not  determined | PCR | Not mentioned | ^26^ |
| 2019 | Portugal | 1 | Birds | Not  determined | MIF | Doxycycline | ^112^ |
| January 2019 to November 2020 | Hunan, China | 44 | Poultry (23)  Pigeons (6)  Unknown (15) | Not  determined | mNGS | Quinolone, or  doxycycline | ^113^ |
| November 2019 to November 2020 | Hunan, China | 16 | Unknown | Not  determined | mNGS | Quinolones or minocycline | ^114^ |
| January 2019 to November 2021 | Guangdong, China | 3 | Unknown | Not  determined | mNGS | Doxycycline | ^115^ |
| December 2019 to January 2020 | Zhejiang,  China | 2 | Chicken | A | mNGS | Moxifloxacin | ^116^ |
| January 2020 | Guangdong,  China | 3 | Unknown | Not  determined | mNGS | Doxycycline | ^117^ |
| October 2020 | Hefei,  China | 1 | Poultry | Not  determined | mNGS | Doxycycline | ^118^ |
| October 2020 | Hunan,  China | 1 | Poultry | Not  determined | mNGS | Doxycycline, meropenem | ^119^ |
| October 2020 | Beijing,  China | 1 | Chicken | Not  determined | mNGS | Moxifloxacin | ^120^ |
| November 2020 | Liaoning,  China | 1 | Unknown | Not  determined | mNGS | Tigecycline | ^121^ |
| December 2020 | Beijing,  China | 1 | Chicken | Not  determined | mNGS | Minocycline, azithromycin | ^120^ |
| December 2020 | Shandong, China | 17 | Ducks | Not  determined | mNGS, qPCR | Doxycycline | ^16^ |
| December 2020 | Chongqing, China | 3 | Parrots | Not  determined | mNGS | Minocycline | ^88^ |
| January 2020 to December 2021 | Guangdong,  China | 30 | Chicken | Not  determined | mNGS | Doxycycline | ^122^ |
| April 2020 to June 2021 | Zhejiang, China | 32 | Poultry (7)  Unknown (25) | Not  determined | mNGS | Doxycycline | ^87^ |
| March 2021 | Beijing,  China | 1 | Birds | Not  determined | mNGS | Minocycline, azithromycin | ^123^ |
| June 2021 | Zhejiang, China | 1 | Chicken | Not  determined | mNGS | Doxycycline, moxifloxacin | ^124^ |
| August 2021 | Zhejiang, China | 1 | Unknown | Not  determined | mNGS | Moxifloxacin | ^125^ |
| 2021 | Guangdong,  China | 1 | Unknown | Not  determined | mNGS | Doxycycline, moxifloxacin | ^96^ |
| 2021 | Rotterdam, The Netherlands | 1 | Birds | Not  determined | PCR | Doxycycline, ciprofloxacin | ^83^ |
| 2021 | Guangdong,  China | 4 | Birds | Not  determined | mNGS | Doxycycline, or quinolones | ^84^ |
| 2021 | Hunan,  China | 5 | Poultry (3)  Unknown (2) | Not  determined | mNGS | Doxycycline | ^95^ |
| 2021 | Tokyo,  Japan | 1 | Pigeons | Not  determined | MIF | Minocycline | ^126^ |
| 2021 | Zhejiang, China | 4 | Poultry | Not  determined | mNGS | Unknown | ^127^ |
| November 2021 to January 2022 | Zhejiang, China | 4 | Geese | E/B | mNGS | Doxycycline | ^128^ |
| December 2021 to September 2022 | Zhejiang, China | 6 | Poultry (4)  Unknown (2) | Not  determined | mNGS | Doxycycline | ^129^ |
| March 2022 | Guangdong,  China | 1 | Chicken | Not  determined | mNGS | Omadacycline | ^97^ |
| April 2022 | Zhejiang,  China | 1 | Parrots | A | mNGS | Moxifloxacin, Azithromy | ^130^ |
| December 2022 | Britain | 2 | Lambs | Not  determined | mNGS | Doxycycline | ^131^ |
| 2022 | Fujian,  China | 2 | Poultry | Not  determined | mNGS | Doxycycline | ^132^ |
| 2022 | Hubei,  China | 1 | Ducks | Not  determined | mNGS | Moxifloxacin | ^133^ |
| 2022 | Hangzhou,  China | 6 | Poultry or birds (5)  Unknown (1) | B | mNGS | Doxycycline | ^134^ |
